# Unusual β1-4-galactosidase activity of an α1-6-mannosidase from *Xanthomonas manihotis* in the processing of branched hybrid and complex glycans

**DOI:** 10.1016/j.jbc.2022.102313

**Published:** 2022-07-31

**Authors:** Yi-Min She, Kody Klupt, Grayson Hatfield, Zongchao Jia, Roger Y. Tam

**Affiliations:** 1 Centre for Biologics Evaluation, Biologic and Radiopharmaceutical Drugs Directorate, Health Canada, Ottawa, Ontario, Canada; 2 Department of Biomedical and Molecular Sciences, Queen's University, Kingston, Ontario, Canada

**Keywords:** *Xanthomonas manihotis* α1-6-mannosidase, β1-4-galactosidase, branched glycans, glycoside hydrolase family 125, structural modeling, glycan structure, dMNJ, deoxymannojirimycin, ER, endoplasmic reticulum, FA, formic acid, GH, glycoside hydrolase, HPAEC-PAD, high pH anionic exchange chromatography with pulsed amperometric detection, MOE, molecular operating environment, MS/MS, tandem mass spectrometry, MQ, Milli-Q, PDB, Protein Data Bank, PGC, porous graphitic carbon

## Abstract

Mannosidases are a diverse group of glycoside hydrolases that play crucial roles in mannose trimming of oligomannose glycans, glycoconjugates, and glycoproteins involved in numerous cellular processes, such as glycan biosynthesis and metabolism, structure regulation, cellular recognition, and cell–pathogen interactions. Exomannosidases and endomannosidases cleave specific glycosidic bonds of mannoside linkages in glycans and can be used in enzyme-based methods for sequencing of isomeric glycan structures. α1-6-mannosidase from *Xanthomonas manihotis* is known as a highly specific exoglycosidase that removes unbranched α1-6 linked mannose residues from oligosaccharides. However, we discovered that this α1-6-mannosidase also possesses an unexpected β1-4-galactosidase activity in the processing of branched hybrid and complex glycans through our use of enzymatic reactions, high performance anion-exchange chromatography, and liquid chromatography mass spectrometric sequencing. Our docking simulation of the α1-6-mannosidase with glycan substrates reveals potential interacting residues in a relatively shallow pocket slightly differing from its homologous enzymes in the glycoside hydrolase 125 family, which may be responsible for the observed higher promiscuity in substrate binding and subsequent terminal glycan hydrolysis. This observation of novel β1-4-galactosidase activity of the α1-6-mannosidase provides unique insights into its bifunctional activity on the substrate structure-dependent processing of terminal α1-6-mannose of unbranched glycans and terminal β1-4-galactose of hybrid and complex glycans. The finding thus suggests the dual glycosidase specificity of this α1-6-mannosidase and the need for careful consideration when used for the structural elucidation of glycan isomers.

Exo-α-mannosidases are mannoside-degrading enzymes found in both prokaryotes and eukaryotes that specifically cleave α1-2, α1-3, or α1-6 glycosidic linkages of terminal mannosyl residues at the nonreducing end and comprise a wide range of glycoside hydrolase (GH) families such as GH31, GH38, GH47, GH63, GH76, GH92, GH99, and GH125, as classified by carbohydrate-active enzymes (1, 2, 3). In the eukaryotic glycan biosynthetic pathway of glycoproteins, mannosyl glycan trimming is initiated in the endoplasmic reticulum (ER) and is crucial for glycoprotein quality control, folding, and ER-associated degradation (4). Sequential processing of glycans by α-mannosidases, *N*-acetylglucosaminyltransferases, *N*-acetylglucosaminidases, and fucosyltransferases form hybrid and complex glycans in the *cis*-, medial-, and *trans*-Golgi apparatus, to impart the glycoprotein with proper protein folding, conformational stability, transport, and bioactivity of mature proteins (5, 6). Elucidation of catalytic mechanisms and interactions of mannosidases is important for understanding various biological processes such as glycan metabolism, structure regulation, cell–cell interactions, and host–pathogen interactions and thus requires extensive knowledge of substrate specificity and cellular localization of individual glycoside hydrolases and glycosyltransferases (7).

In addition to their biological significance, mannosidases and other glycosidases have become valuable reagents to aid in characterizing glycan structures in glycoprotein-based therapeutics, mapping glycan epitope–receptor interactions (8), and in glycan chemical semisynthesis (9, 10, 11, 12). Unlike the characterization of proteins and oligonucleotides, analysis of glycans and their isomeric structures can be difficult to achieve due to structural complexities, dynamic regulation, and nontemplated biosyntheses. Glycan modifications that are incorporated into glycoproteins are diverse and are dependent on the host species and their aforementioned endogenous glycosylation machinery. High mannose *N*-glycans comprise a class of glycans commonly found in biotherapeutic glycoproteins, and their presence has been associated with increased serum clearance (13); they are also capable of binding to endogenous mannose-binding lectin proteins/receptors and DC-SIGN receptors that are present on the surface innate immune cells (14). With the growing appreciation of the importance of glycosylation in protein bioactivity, novel glycoengineering techniques and varying cell expression systems such as plant and yeast are being explored in biotherapeutic production, which may produce diverse glycan structures (15, 16, 17). For example, yeast systems can produce high-mannosylated species, while plants can also produce these, along with hybrid structures that may also contain nonhuman epitopes such as core α1-3 fucose and bisecting β1-2-xylose glycans (17, 18). Moreover, changes in manufacturing conditions and upregulation of specific glycosyltransferase genes in glycoengineering processes can produce unexpected glycan structures by creating an imbalance in the glycosylation machinery cascade within the ER and Golgi apparatus (19). Given the potential impact of glycosylation on the bioactivity and stability of a glycoprotein, it is important that the glycan structures are accurately characterized in biotherapeutic drugs. To tackle this challenge, glycosidase enzymes that cleave specific glycan bonds are commonly used in glycan structure characterization (20). PNGaseF is amongst the most widely used enzyme that specifically cleaves *N*-glycans from asparagine residues of glycoproteins (20), liberating intact glycans that can be directly analyzed by chromatographic techniques. Cleavage of mannose-mannose residues with specific glycosidic linkages can also be achieved using various mannosidases (21), in particular, unbranched mannosyl-α1-6-mannosyl (Man-α1-6-Man) residues can be cleaved by α-1-6-mannosidase (21, 22), and has been used in identifying unbranched Man-α1-6-Man glycosyl linkages in biological samples (23) and in glycan semisynthesis (12, 24). Previous literature reported that α1-6-mannosidase cloned from *Xanthomonas manihotis* is unable to cleave Gal-β1-4GlcNAc residues in linear oligosaccharides, as determined by TLC of linear oligosaccharide substrates labeled with a 7-amino methylcoumarin tag (21).

In our efforts to map Man-α1-6-Man epitopes in *N*-glycan structures using α1-6-mannosidase from *X. manihotis*, we serendipitously observed cleavages of branched *N*-glycans containing a terminal β1-4-galactose residue at the nonreducing end. Herein, we report this unexpected reactivity where, in addition to its native α1-6-mannosidase activity on unbranched glycans, Gal-β1-4-GlcNAc linkages in branched *N*-glycans are cleaved by this enzyme. Using docking simulation studies, we highlight potential interactions that may be responsible for this unexpected reactivity. The observations provide unique insights into the dual glycosidase activity of the *X. manihotis* α1-6-mannosidase toward cleavages of unbranched and branched glycans, including selective recognition and degradation of terminal mannose and galactose residues in glycan substrates by the bacterial enzyme. Our findings show that careful structural elucidation needs to be considered when using glycosidase enzymes for mapping glycan structures, particularly in complex mixtures of biological samples that have a plethora of complex, hybrid, and high mannose *N*-glycans. However, our results do not detract from its usefulness in reacting with more defined high mannose glycan mixtures and rather suggest it may be an additional useful tool in the synthesis of monogalactosylated branched glycans.

## Results

### β1-4-galactosidase activity of *X. manihotis* α1,6-mannosidase in the processing of branched glycans

**Figure 1:**
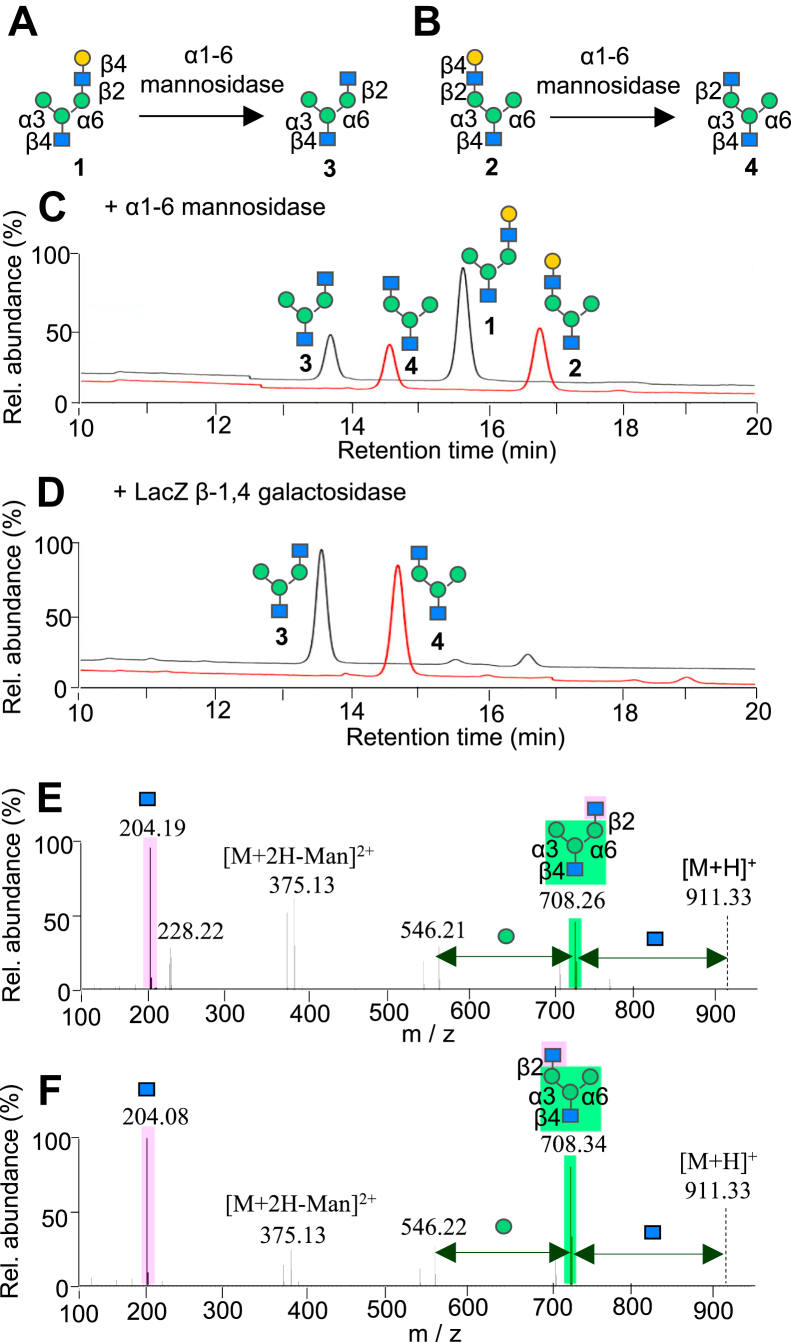
**Unexpected β1-4-galactosidase reactivity of *X. manihotis* α1-6-mannosidase with branched monogalactosylated glycans.***A* and *B*, reaction schemes of branched monogalactosylated glycan isomers 1 and 2 (100 μM) with α1-6-mannosidase from *X. manihotis* (6 U/μl, 20 μl, 37 °C) to form unexpected degalactosylated products. *C* and *D*, HPAEC-PAD chromatograms of the monogalactosylated glycans reacted with α1-6-mannosidase or LacZ β1-4-galactosidase, showing the formation of a hydrolyzed product at approximately 14 min. *E* and *F*, MS/MS spectra of the doubly charged ions of the degraded products showing the loss of a branching side chain GlcNAc residue to form the diagnostic ion at m/z 204.08 and its complementary fragment [(M-GlcNAc+H)^+^] at m/z 708.26, indicating that the GlcNAc residue is localized at the terminal position after degalactosylation by α-1-6-mannosidase. HPAEC-PAD, high pH anionic exchange chromatography with pulsed amperometric detection.

We initially observed that treatments of pseudohybrid and hybrid glycans (compounds 1 and 2 in Fig. 1, *A* and *B*, respectively) with α1-6-mannosidase from *X. manihotis* yielded degraded products with a shift of elution times that were observed by high pH anionic exchange chromatography with pulsed amperometric detection (HPAEC-PAD) analyses (Fig. 1*C*). The term pseudohybrid refers glycans comprising a GlcNAc residue attached to the mannose of the α6 antenna, whereas hybrid refers those comprising a GlcNAc on the α3 antenna (25). The potential cleavage of the branched Man-α1-6-Man linkage in hybrid glycan 2, that is, the Man-α1-6-Man glycosidic bond of the exposed monomannose residue on the α6-antenna, was ruled out as we also observed the simultaneous presence of a proteolytic product from pseudohybrid glycan 1, which does not contain any Man-α1-6-Man linkages. To understand where the cleavage occurred, we treated the two monogalactose-containing branched glycans 1 and 2 with LacZ β-galactosidase to selectively cleave terminal galactose-β1-4-GlcNAc residues. HPAEC-PAD analyses of these reactions revealed peaks that have the same retention times as those produced by α1-6-mannosidase for both glycans 1 and 2 (Fig. 1*D*). To further confirm the consistency of the glycan structures, we analyzed the α1-6-mannosidase reactions by porous graphitic carbon (PGC)–LC-MS/MS, an elegant method that is able to resolve the branch-specific linkage structures of α- and β-anomers of each glycan (26). The β- and α-anomers of the degalactosylated products were observed at earlier retention times compared to those of the starting materials (Fig. S1, *C* and *D*). Tandem mass spectrometry (MS/MS) measurements of the glycan precursors 1 and 2 show the presence of the high abundance diagnostic ion at m/z 366.17 ([GalGlcNAc+H]^+^) and the complementary fragment at m/z 708.28 ([M-365+H]^+^) from its molecular parent ion, indicative of the glycan branch-specific fragmentations (Fig. S1, *E* and *F*) (26, 27). Enzymatic reactions of branched glycans 1 and 2 (*i.e.*, the protonated molecule at m/z 1073.38 ([M+H]^+^)) with α1-6-mannosidase show the formation of glycan products at m/z 911.34, indicating the loss of a hexose residue (m/z 162.04). MS/MS analyses of the products at m/z 911.34 reveal the branch-specific diagnostic ion at m/z 204.19 or m/z 204.08 ([GlcNAc+H]^+^), as well as the complementary fragment at m/z 708.34 ([M-GlcNAc+H]^+^) (Fig. 1, *E* and *F*), indicating glycan structures containing a terminal GlcNAc residue at the branched side chain, that is, following the unexpected loss of a β-1-4-galactose residue at the terminal branch upon reaction with α1-6-mannosidase.

**Figure 2:**
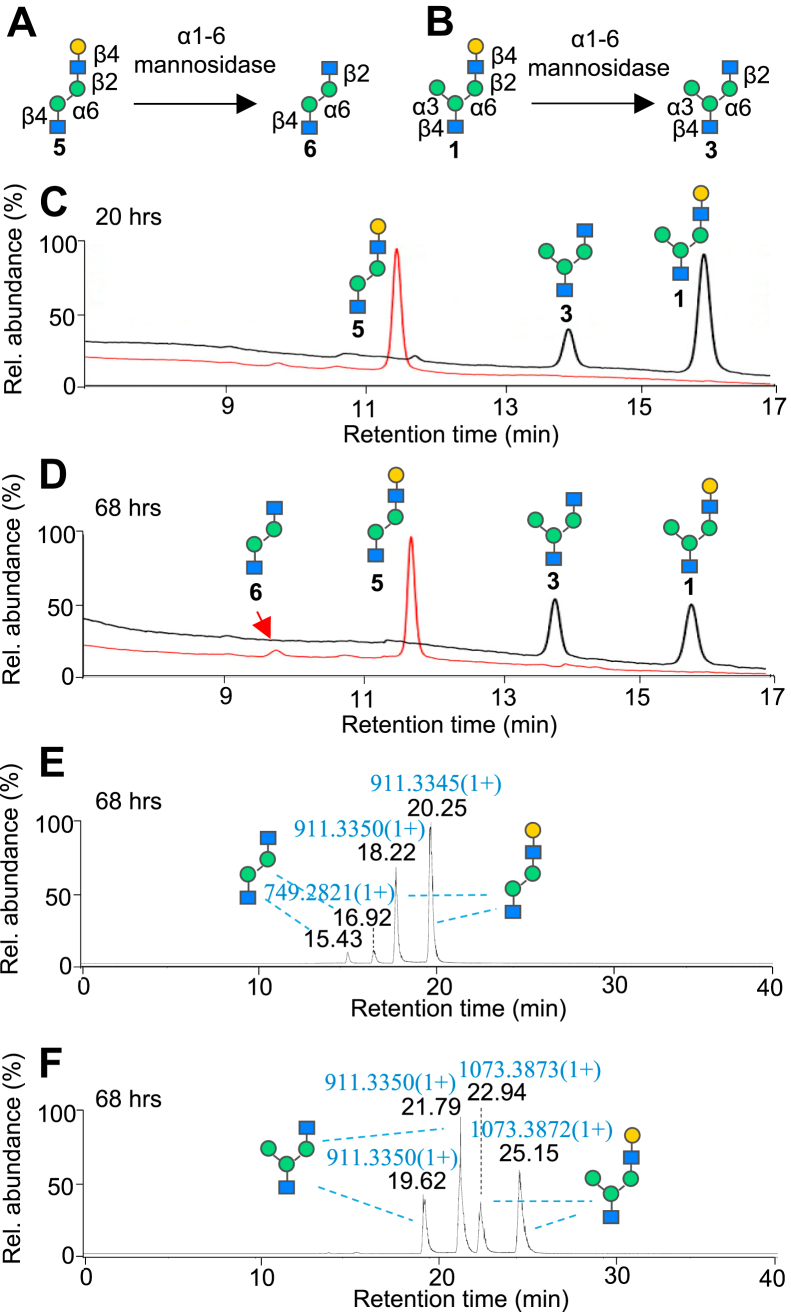
**Parallel comparison of enzymatic reactions of linear and branched monogalactosylated glycans with *X. manihotis* α1-6-mannosidase reveals selectivity toward branched glycans.***A* and *B*, reaction schemes of linear Gal-β1-4-GlcNAc-containining glycan 5 and branched Gal-β1-4-GlcNAc-containing glycan 1 with α1-6-mannosidase. *C* and *D*, overlaid HPAEC-PAD chromatograms of degalactosylated product peaks from the branched glycan (*black traces*) and linear glycan (*red traces*) after reactions for 20 h and 68 h. *E* and *F*, total ion chromatograms of the reactions of linear glycan and branched glycan after 68 h, analyzed by PGC-LC-MS. Greater cleavage efficiency of the Gal-β1-4-GlcNAc bond is observed in the branched glycan 1 compared to the linear glycan 5. Enzymatic reactions were performed in parallel on 100 μM of each glycan with 2 U/μl of α1-6-mannosidase at 37 °C. HPAEC-PAD, high pH anionic exchange chromatography with pulsed amperometric detection; PGC, porous graphitic carbon.

To further validate that the loss of a hexose residue was not caused by the cleavage of the monomannose residue at the antennal position, we treated pseudohybrid glycan 1 with α1-2,3-mannosidase to cleave the α3-antennal monomannose residue to form the linear pentasaccharide 5 (Fig. S2). Analysis by HPAEC-PAD shows the presence of a peak at the earlier retention time of 11.5 min (Fig. S2*C*) compared to that of the degalactosylated pseudohybrid glycan 3 at 13.7 min (Fig. 2*C*). In addition, PGC-LC-MS/MS reveals a different fragmentation pattern of the demannosylated product at m/z 911.33 compared to that of the degalactosylated glycan at m/z 911.33 following treatment with α1-6-mannosidase (Fig. S2*G versus* Fig. 1*E*). High abundance peaks at m/z 546.14 ([M-GalGlcNAc+H]^+^) along with its diagnostic ion at m/z 366.18 ([GalGlcNAc+H]^+^) are observed for the unbranched glycan 5 containing a terminal GalGlcNAc (Fig. S2*G*).

In light of this unexpected activity, we questioned whether the enzyme contained impurities that resulted in potential β1-4-galactosidase activity. To rule out contamination that may have occurred during protein expression, SDS-PAGE of α1-6-mannosidase was performed, and the gel image shows merely a clear band at approximately 47 kDa and no other protein on the gel (Fig. S3). Further examination was conducted by highly sensitive Orbitrap LC-MS/MS analyses of the protein digests using trypsin and chymotrypsin separately as indicated later, followed by searches against NCBI and Uniprot databases; the only protein identified was *X. manihotis* α1-6-mannosidase.

### Confirmed mannosidase activity of *X. manihotis* α1-6-mannosidase on paucimannose

Next, we confirmed the known function of this enzyme by testing for its inherent α1-6-mannosidase activity by reacting it with paucimannose (Man_3_GlcNAc_2_) in combination with or without α1-2,3 mannosidase (Fig. S4). As α1-6-mannosidase only cleaves linear α1-6-mannose linkages, α1-2,3 mannosidase was introduced to remove the branched α1-3 monomannose residue of the trimannosyl core to form the linear glycan. In the absence of α1-2,3 mannosidase, HPAEC-PAD and PGC-LC-MS analyses show that no cleavage occurs on paucimannose when reacted with α1-6-mannosidase alone; the chromatogram retains the same peak as the starting Man_3_GlcNAc_2_ (*red* and *black* traces, Fig. S4*A*), consistent with the PGC-LC-MS β- and α-anomeric peaks at m/z 911.3343 for ([Man_3_GlcNAc_2_+H]^+^) (Fig. S4*B*). Treatment with α1-2,3 mannosidase alone results in the formation of a new peak at 7.3 min by HPAEC-PAD analysis (*blue* trace, Fig. S4*A*), which is further confirmed by the PGC-LC-MS β- and α-anomeric peaks at m/z 749.282 ([Man_2_GlcNAc_2_+H]^+^, Fig. S4*D*). Combination of both enzymes results in the expected cleavage of two mannose residues to form the ManGlcNAc_2_ trisaccharide at 5.5 min (*purple* trace, Fig. S4*A*) and is confirmed by the presence of the PGC-LC-MS peaks at m/z 587.229 ([ManGlcNAc_2_+H]^+^, Fig. S4*C*).

Taken together, our initial data suggest that this unexpected β1-4-galactosidase activity of the α1-6-mannosidase may be highly correlated to the structural characteristics of branched glycans. Previous literature reported that α1-6-mannosidase from *X. manihotis* does not cleave linear Gal-β1-4-GlcNAc-containing substrates such as Gal-(±Fucα1-3)-β1-4-GlcNAc-β1-3-Gal-β1-4 Glc label-ed with 7-amino methylcoumarin after 4 h (for afucosylated oligosaccharide) or 20 h (for fucosylated oligosaccharide) at 37 °C, as analyzed by TLC (21). To confirm this observation, we tested the β1-4-galactosidase activity of α1-6-mannosidase on a linear pentasaccharide 5 prepared *via* cleavage of the α3 antenna of the pseudohybrid glycan 1 using treatment α1-2,3 mannosidase (Fig. S2*A*), followed by PGC-HPLC purification. Treatments of 100 μM of either the linear (Fig. 2*A*) or branched (Fig. 2*B*) glycan 1 with α1-6-mannosidase (2 U/μl) resulted in 23% cleavage of the latter branched glycan 1 but less than 3% of the linear saccharide 5 after 20 h at 37 °C (Fig. 2*C*), as determined by HPAEC-PAD analysis. Further extended incubation to 3 days at 37 °C resulted in slightly higher (7%) degalactosylation of the linear structure, whereas 55% cleavage was achieved in the branched pseudohybrid structure by HPAEC-PAD analysis (*black* trace, Fig. 2*D*) and is confirmed by PGC-LC-MS analysis (Fig. 2, *E* and *F*).

### Structure-dependent cleavages of complex glycans *by X. manihotis* α1-6-mannosidase

**Figure 3:**
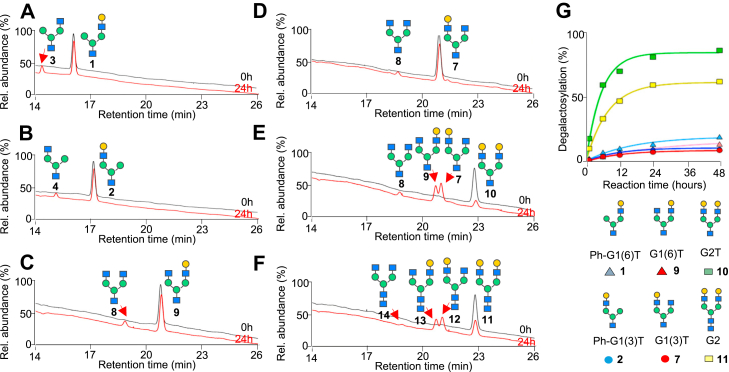
***X. manihotis* α1-6-mannosidase has increased β1-4-galactosidase activity toward digalactosylated complex glycans and slight selectivity toward the α6 antenna.** Cleavage of Gal-β1-4-GlcNAc in glycan substrates using α1-6-mannosidase: (*A* and *B*) branched glycans containing single Gal-β1-4-GlcNAc branch at the α6 antenna 1 or α3 antenna 2; (*C* and *D*) biantennary complex glycan containing a terminal galactose at the α6 antenna 9 or α3 antenna 7; (*E* and *F*) digalactosylated complex glycan (G2) containing a mono-GlcNAc residue at the reducing end 10 or a di-GlcNAc chitobiose core at the reducing end 11. *G*, time-course of degalactosylation reactions of branched glycans (100 μM) with 2 U/μl of α1-6-mannosidase at 37 °C, showing an increase in degalactosylation in digalactosylated complex glycans.

Having demonstrated the selective β1-4-galactosidase activity of *X. manihotis* α1-6-mannosidase toward branched pseudohybrid glycan 1 over linear glycan 5, we next expanded the scope of the glycan structural requirements on complex biantennary glycans. We targeted the influence of the carbohydrate residues on the agalactosylated antenna (*i.e.*, terminal monomannose-containing [(pseudo)hybrid-type] *versus* terminal GlcNAc-containing residue [complex-type glycans] of the agalactosylated antenna), as well as the antenna specificity of the terminal Gal-β1-4-GlcNAc residue (*i.e.*, α3 antenna *versus* α6 antenna) (Fig. 3). Treatment of equimolar (100 μM) truncated (*i.e.*, containing only one reducing end GlcNAc residue) complex or (pseudo)hybrid glycans containing a single terminal galactose residue on either the α3 or α6 antenna (compounds 1, 2, 7, 9) with α1-6-mannosidase (2 U/μl) revealed that the location of the terminal monogalactose residue has a slightly greater effect on its cleavage compared to the presence of a terminal monomannose or a GlcNAc-β1-2-mannose residue on the opposite agalactosylated antenna (Fig. 3). The terminal galactose on the α3 antenna (compounds 2 and 7, circle data points in Fig. 3*G*) were generally less susceptible to cleavage (9%–11% after 48 h) compared to the α6 antenna (compounds 1 and 9, 14%–20% after 48 h, triangle data points in Fig. 3*G*). There were minimal differences between hybrid 2 and complex glycan 7 when galactose is on the α3 antenna (*red versus blue* circle data points, Fig. 3*G*), but interestingly, a slight increase in cleavage of the pseudohybrid glycan containing the galactose on the α6 antenna 1 is observed compared to its complex glycan analog 9 (*blue versus red* triangle data points in Fig. 3*G*).

Surprisingly, treatments of the same concentrations of enzyme (2 U/μl) with digalactosylated substrates (10 and 11, 100 μM) resulted in a greater cleavage of the terminal β1-4-galactose residue than any of the monogalactosylated glycans (Fig. 3*E*). After 24 h, degalactosylation of the truncated digalactosylated 10 glycan reached a reaction yield of 83%, compared to monogalactosylated glycans (compounds 1, 2, 7, 9) that are at a range of 8% to 14%. Cleavage of the nontruncated (*i.e.*, bearing two GlcNAc residues at the reducing end) digalactosylated glycan 11 yielded similar results (Fig. 3*F*), albeit at a lower degalactosylation yield (60% at 24 h) than truncated glycan 10. Examining the cleavage products shows that the relative proportions of the two monogalactose glycoforms are formed at a similar ratio, with a slightly higher amount of the α3 glycoforms produced (7 and 12); this is consistent with our observation that the α6 analog is slightly more susceptible to β1-4-galactose cleavage by this α1-6-mannosidase compared to the α3 analog (Fig. 3*E*).

**Figure 4:**
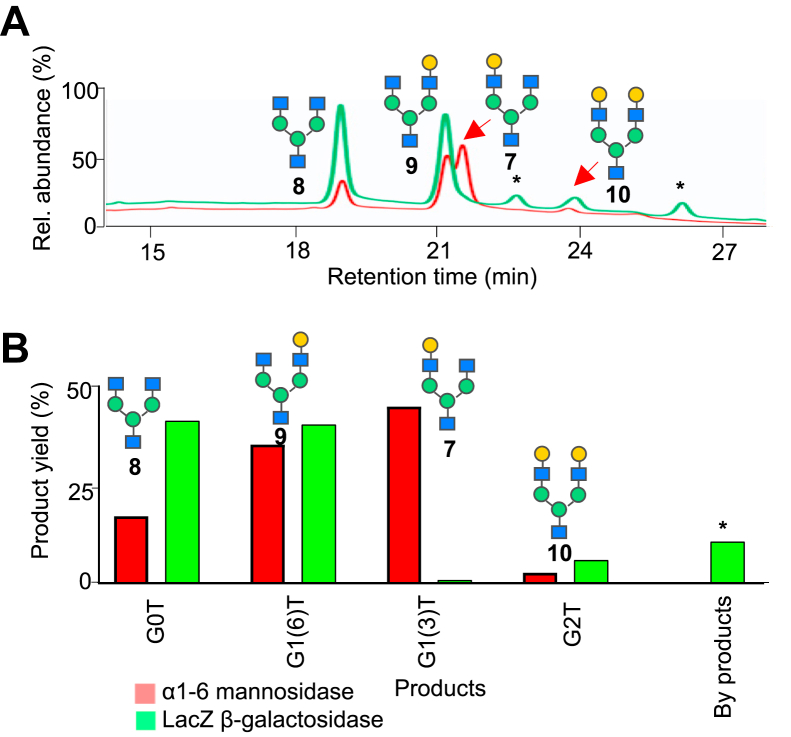
**Comparison of β1-4-galactosidase activity between α1-6-mannosidase and LacZ β1-4-galactosidase.***A*, overlaid HPAEC-PAD chromatogram traces showing enzymatic reactions of digalactosylated G2T glycan with LacZ β1-4-galactosidase (*green trace*) or α1-6-mannosidase (*red trace*). *B*, comparison of product distributions between α1-6-mannosidase and LacZ β1-4-galactosidase at approximately 95% completion of the reactions. HPAEC-PAD, high pH anionic exchange chromatography with pulsed amperometric detection.

Notably, the distribution of degalactosylated products are in contrast to β1-4-galactose cleavage with LacZ β1-4-galactosidase, in which the latter has increased reactivity toward cleaving the Gal-β1-4-GlcNAc linkage in the α3 antenna than the α6-antenna (Fig. 4, *A* and *B*), consistent with literature (28, 29). To further compare the relative β1-4-galactosidase activity between α1-6-mannosidase and LacZ β1-4-galactosidase, we examined the cleavage products at approximately 95% reactivity of the initial digalactosylated glycan to monitor cleaved intermediates. We observe only 1% formation of the monogalactosylated glycan at the α3 antenna using LacZ β1-4-galactosidase, while 45% is formed using α1-6-mannosidase. However, an opposite trend is observed with the monogalactosylated glycan at the α6 antenna (45% *versus* 35% for LacZ *versus* α1-6-mannosidase). Interestingly, there is less formation of the agalactosylated glycan 8 (17% G0) when treated with the α1-6-mannosidase compared to LacZ β1-4-galactosidase (39% G0) (Fig. 4*B*).

To determine whether the unexpected β1-4-galactosidase activity was due to high concentrations of glycan concentrations used in our study (100 μM), we performed a concentration scan of the digalactosylated glycan substrate 10, ranging from 50 to 0.050 μM. We observe that the relative hydrolysis increased as the glycan concentrations decreased (Fig. S5).

Next, we compared the relative activities of α1-6-mannosidase to cleave either its native Man-α1,6-Man substrate or the unexpected Gal-β1-4-GlcNAc moiety in a branched glycan substrate by treating varying concentrations of α1-6-mannosidase with 25 μM of linear Man-α1-6-Man-GlcNAc_2_ tetrasaccharide or truncated digalactosylated G2T glycan 10, respectively. As expected, a higher enzyme concentration was required for β1-4-galactosidase activity compared to the native α1-6-mannosidase activity; to achieve approximately 40% hydrolysis in 2 h, 2 U/μl of α1-6-mannosidase was required to cleave the Gal-β1-4-GlcNAc linkage from the G2T glycan 10, while only 0.050 U/μl was required to cleave a similar amount of the native Man-α1-6-Man linkage from a Man-α1-6-Man-GlcNAc_2_ tetrasaccharide (Fig. S6). However, at longer time points, α1-6 mannosidase activity was decreased at lower concentrations, possibly due to enzymatic degradation.

### Inhibition of α1-6-mannosidase and β1-4-galactosidase activities using 1-deoxymannojirimycin

To demonstrate that the same active site may be responsible for the glycosidase activity of both α1-6-mannose and Gal-β1-4-GlcNAc, we performed enzymatic reactions in the presence of an α-mannosidase-specific inhibitor 1-deoxymannojirimycin (dMNJ) (30), which has been shown by X-ray crystallography to bind within the active site of SpGH125 (Protein Data Bank [PDB] 3QRY), a similar α1-6-mannosidase of the GH125 family (31). Using a linear Man-α1-6-Man-GlcNAc_2_ tetrasaccharide or the digalactosylated G2T glycan 10 as glycan substrates, we observed a dose-dependent inhibition of mannosidase or galactosidase activity of *X. manihotis* α1-6-mannosidase, respectively. At ratio of 10 nmol dMNJ per unit of α1-6-mannosidase, we observed approximately 75% inhibition of both α1-6-mannosidase and β1-4-galactosidase activity (*red versus green* traces in Fig. S7, *A* and *B*), and treatment with 100 nmol dMNJ per unit of α1-6 mannosidase completely inhibited activity (Figs. S7, *A* and *B* and S8). We further confirm the specificity of this inhibition by treating G2T glycan 10 with 10 or 100 nmol dMNJ inhibitor per unit of enzyme of Lac Z β1-4-galactosidase. At these dMNJ concentrations, we did not observe any inhibition of β1-4-galactosidase activity by LacZ after 4 h of reaction (Fig. S7*C*). Together, these data suggest that the action of dual enzymatic activity may involve the same binding site to glycan substrates within the catalytic region of *X. manihotis* α1-6-mannosidase.

### *X. manihotis* α1-6-mannosidase is a glycosidase in the GH family 125

**Figure 5:**
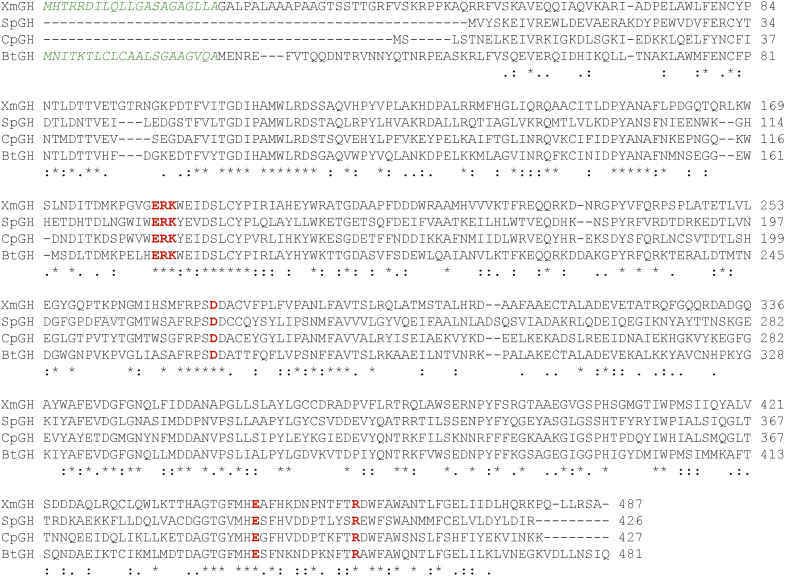
**Protein sequence alignment of α1-6-mannosidases in the GH125 family.** Multiple sequence alignment is generated using the Clustal Omega program (https://www.ebi.ac.uk/Tools/msa/clustalo/). XmGH125: *Xanthomonas manihotis* α1-6-mannosidase GH125, NCBI accession WP_017155573.1; SpGH125: *Streptococcus pneumoniae* α1-6-mannosidase GH125, UniProKB accession A0A0H2URZ6, PDB code 3QRY; CpGH125: *Clostridium perfringens* α1-6-mannosidase GH125, UniProKB accession Q8XNB2, PDB code 2NVP; BtGH125: *Bacteroides thetaiotaomicron* α1-6-mannosidase GH125, UniProKB accession #Q8A185, PDB code 2p0v. The predicted signal peptides at the protein N terminus are highlighted in *italic green*, and the conserved interacting residues with glycan substrates are highlighted in *bold red*. GH, glycoside hydrolase; PDB, Protein Data Bank.

Reversed phase LC-MS/MS analyses of a tryptic digest of the α1-6-mannosidase followed by Mascot database search identified the recombinant protein sequence in the GH family 125 (NCBI accession # WP_017162505) derived from its precursor protein WP_017155573 from *X. manihotis* (referred herein as XmGH125), in which a 98% sequence coverage of the intact protein was obtained (Table S1). Manual inspection on the data and additional LC-MS/MS measurements of a chymotrypsin digest confirmed the full-length protein sequence identification except two residues at the protein C terminus. In addition, several tryptic peptide fragments were found at the ragged protein N termini. MS/MS fragmentations on the peptide at m/z 897.9701 showed the presence of a possible peptide VALPALAAAPAAGTSSTTGR of the N-terminal protein (residues 5–24, WP 017162505). However, mass accuracy measurements indicate the residue at the peptide N terminus is acetylated glycine (57.0215 + 42.0106 = 99.0321 units) rather than its isobaric valine (99.0684 units), resulting in mass errors of −1.25 ppm and −22.83 ppm, respectively (Table S1). The presence of two peptides at m/z 1048.0790 and m/z 906.4841 revealed the N-terminal sequence extension of the protein at residues 1 to 24 and 4 to 24 (WP_017162505). Overall, LC-MS/MS analyses identified the amino acid residues 1 to 468 of the α1-6-mannosidase, as part of its precursor XmGH125 protein (WP_017155573) in which the missing N-terminal 17 to 21 amino acids containing two consecutive arginines may behave as a putative twin-arginine (Tat) signal peptide (Fig. 5) (32). The identified α1-6-mannosidase protein, possessing the β1-4-galactosidase activity on branched glycans, has a theoretical molecular mass of 54 kDa, which is smaller than that of *X. manihotis* β-galactosidase (66 kDa) and other generally large prokaryotic β-galactosidases (∼120–230 kDa) (33, 34). The BLAST search shows no sequence homology to known prokaryotic and eukaryotic β-galactosidases in the NCBI database.

### Molecular basis of XmGH125 α1-6-mannosidase and glycan substrate interactions

To better understand the unexpected β-1,4-galactosidase activity in XmGH125, we first performed the multiple sequence alignment of XmGH125 α1-6-mannosidase (WP_017155573) with homologous GH125 proteins by the Clustal Omega program, which shows a high identity of amino acids that localized at residues 56 to 473 (Figs. 5 and S9). A sequence similarity search of XmGH125 against the reference mannosidases (*i.e.*, PDB codes 3QRY, 2NVP, 2P0V) in the structural PDB data were also conducted using an embedded PDB search function in Molecular Operating Environment (MOE). Note that the 3QRY and 3QSP represent apoenzyme and holoenzyme structures, respectively, of the same α1-6-mannosidase. In particular, XmGH125 α1-6-mannosidase and the reference protein 3QRY are 36.9% identical and 55.1% similar with respect to their residue sequences. Superimposing the two structures, using the alignment results, reveals the two enzymes align with an RMSD of 2.3 Å, indicative of a close alignment with high structural similarity.

Alignment of several closely related mannosidases with XmGH125 highlighted a conserved ERK motif (^128^ERK^130^ in 3QRY, ^129^ERK^131^ in 2NVP, and ^184^ERK^186^ of the XmGH125 α1-6-mannosidase) that was well superimposed (Fig. 5). This motif has been previously identified to be a part of the mannosidase active site that interacts with a mannose residue *via* a hydrogen bonding network in 3QRY and 2NVP (31). Interestingly, a *C*-terminal tryptophan residue beside the ERK motif of XmGH125 is similarly found in 2P0V, while a tryptophan residue is located on the *N*-terminal side of the ERK motif in other mannosidases such as 3QRY and 2NVP. Moreover, residues R405 and E393 of the reference mannosidases 3QRY and 2NVP superimpose well with the R459 and E447 of XmGH125 and has been structurally determined to be involved in coordination of a nucleophilic water in the catalytic domain (31). D274 of XmGH125 is also aligned with D218 of 3QRY and D220 of 2NVP, and D266 of 2P0V, and has been previously proposed to be a catalytic acid by donating a proton to the glycosidic oxygen between the two mannose residues to facilitate disaccharide hydrolysis.

**Figure 6:**
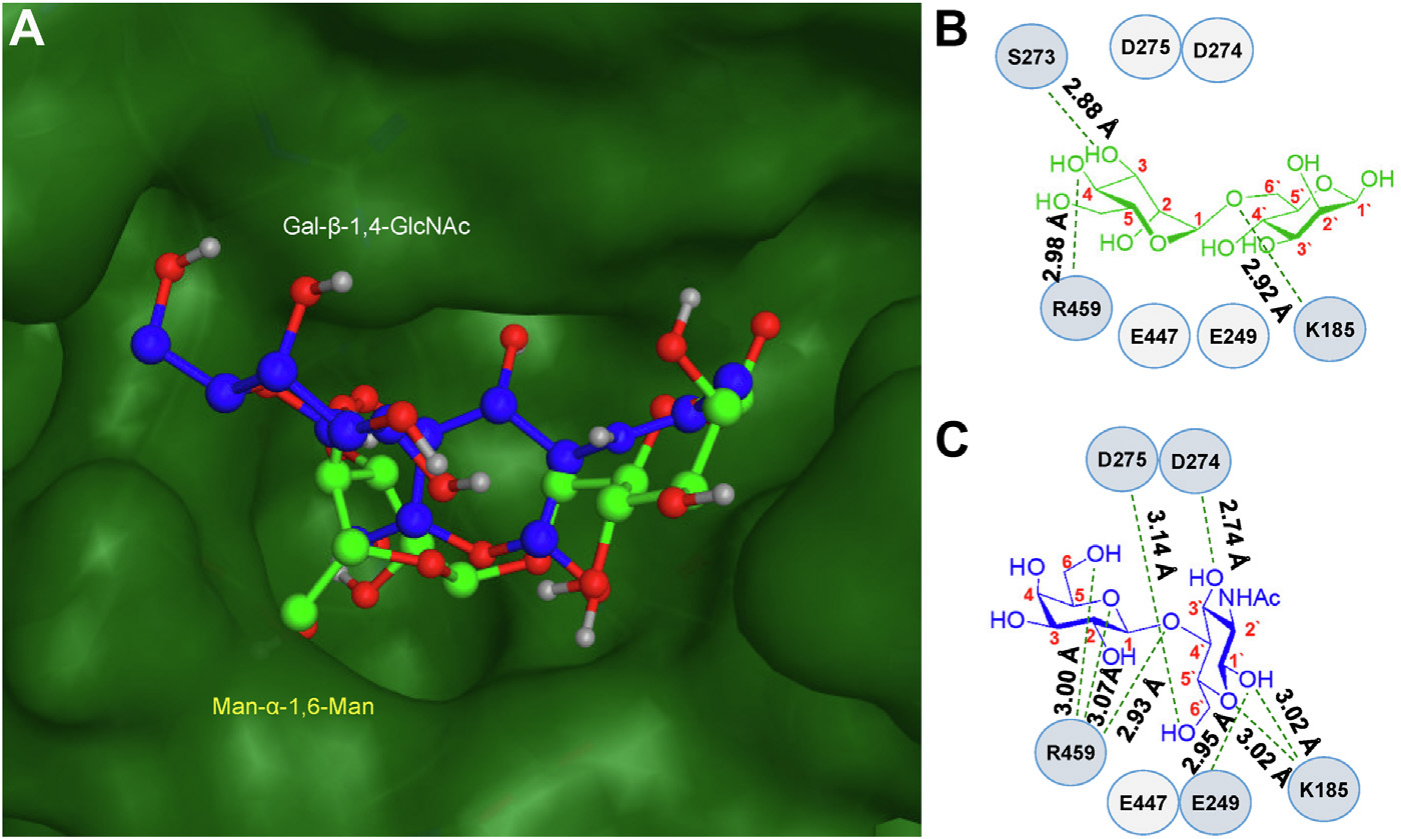
**Docking simulations determine the structural basis of dual catalytic activity.***A*, docking of Man-α1-6-Man (*bright green*) and Gal-β1-4-GlcNAc (*blue*) model disaccharides into the active site of α1-6-mannosidase from *X. manihotis* (XmGH125). XmGH125 protein surface is in *darker green*. *B* and *C*, ligand interaction maps of (*B*) Man-α1-6-Man and (*C*) Gal-β1-4-GlcNAc model disaccharide and XmGH125 residues. *Green dashed lines* indicate side chain and backbone ligand–residue interactions. *B*, Man-α1-6-Man ligand is stabilized by K185, S273, and R459 of XmGH125, while (*C*) Gal-β1-4-GlcNAc is stabilized by K185, E249, D274, D275, and R459 of XmGH125. Docking and figures generated in Molecular Operating Environment 2020, Chemical Computing Group, and BioRender, respectively.

Next, we used postdocking simulation to examine potential glycan–enzyme interactions of XmGH125 α1-6-mannosidase with a truncated α-D-mannopyranosyl-(1→6)-D-mannopyranose (Man-α1-6-Man) or β-D-galactopyranosyl-(1→4)-*N*-acetyl-D-glucosamine (Gal-β1-4-GlcNAc) disaccharides as model ligands (Fig. 6*A*). As a control, we used reference mannosidase 3QRY with its native Man-α1-6-Man containing ligand. Attempts using full-sized polysaccharides including the larger unbranched Man-α1-6-Man glycan (Man_2_GlcNAc_2_) into the reference 3QRY mannosidase were unsuccessful and revealed several different binding modes. Size and electrostatic repulsion of the full polysaccharides made it difficult for the ligand to enter the catalytic pocket of the enzymes, while trimming the ligands to their terminal disaccharide residues improved simulations and reduced electrostatic repulsion and were used for all simulations.

We initially performed postdocking simulation of XmGH125 with its native Man-α1-6-Man ligand, which showed that K185 interacted with the glycosidic oxygen of the ligand by hydrogen bond (distance of 2.92 Å) (Fig. 6*B*). In the reference 3QRY mannosidase, the analogous K130 forms a stabilizing interaction with the C6 hydroxyl group of the nonreducing mannose residue. The Man-α1-6-Man ligand was further stabilized in the catalytic pocket of XmGH125 by R459 and S273, forming additional hydrogen bonds with the peripheral disaccharide hydroxyl groups. In the reference 3QRY, the equivalent and superimposable R405 interacts with the glycosidic oxygen to stabilize the ligand. E447 of XmGH125, analogous to E393 in 3QRY, is distant from the ligand and does not form a direct interaction with the disaccharide; however, previous reports suggest the analogous E393 coordinates with a nucleophilic water molecule followed by an inverting catalytic mechanism that is involved in disaccharide hydrolysis (31). D274 of XmGH125 (which is equivalent to the catalytic acid D218 residue in 3QRY) is also found near the glycosidic oxygen of Man-α1-6-Man, though slightly farther than the D218 in 3QRY, at 5.42 Å for XmGH125 *versus* 4.7 Å for 3QRY.

In contrast, the Gal-β-1,4-GlcNAc disaccharide engages in a different binding mode with the catalytic pocket of XmGH125 (Fig. 6*C*). The Gal-β1-4-GlcNAc glycosidic oxygen forms a hydrogen bond with R459 (at a distance of 2.93 Å) of XmGH125. Peripheral disaccharide hydroxyl groups are further stabilized by several residues including K185, E249, D274, and D275 in the catalytic pocket. Interestingly, R459 also coordinates with the C6 hydroxyl group and the endocyclic pyranose oxygen of the terminal galactose residue. Docking simulations of Gal-β1-4-GlcNAc with the reference mannosidase 3QRY, as expected, did not provide a reliable binding mode.

Using a surface rendering map in MOE, it is identified that neither disaccharide ligands penetrate the surface of the catalytic pocket, indicating that there is no steric clash between the protein and ligand (Fig. 6). A receptor-surface view for the catalytic pocket of XmGH125 generated in MOE reveals a shallow ligand pocket (Fig. S10*A*). In this shallow binding pocket, residue R459 is in an extended conformation to form contact with either disaccharide, and a loop containing an extended M407 is positioned toward the binding pocket. This shallow configuration of key residues in the binding pocket prevents either disaccharide ligand from penetrating the protein surface in the docking simulation.

In contrast, a different mode of ligand binding is observed in the control reference mannosidase 3QRY (Fig. S10*B*). Here, the Man-α1-6-Man ligand exhibits a binding mode in which the glycosidic oxygen interacts *via* hydrogen bonding with the R405 (R459 in XmGH125) at the bottom of the binding pocket. In contrast to the Man-α1-6-Man binding mode observed with XmGH125, the ligand when positioned in the reference mannosidase binding pocket appears to deeply penetrate the enzyme surface. Here, residue R405 is in a collapsed conformation, and the disordered loop proximal to the catalytic site is observed to rotate away from the ligand. A monosaccharide unit of the Man-α1-6-Man ligand gains the ability in the docking simulation to situate itself deep into the narrow pocket of the reference mannosidase 3QRY.

## Discussion

Glycosidase enzymes are useful reagents in characterizing glycan structures, glycan synthesis, and examining glycan epitopes in glycan ligand-receptor binding (8, 9, 10, 11). Enzyme specificity for ligands is important to understand and characterize to ensure that the resulting products are correct, especially in the context of the complex biological samples. Glycan characterization is especially difficult due to their nontemplated biosynthesis and dynamic regulation by intracellular and extracellular glycosyltransferases and glycosidase enzymes. Our results herein demonstrate that α1-6-mannosidase isolated from *X. manihotis* (*i.e.*, XmGH125), in addition to its ability to cleave unbranched Man-α1-6-Man linkages, is capable of cleaving Gal-β1-4-GlcNAc linkages in branched (pseudo)hybrid and complex glycans. However, β1-4-galactosidase activity of the α1-6-mannosidase was unfavored in unbranched glycans, consistent with the literature (21). It is worth noting that the XmGH125 α1-6-mannosidase shows no sequence homology to any known prokaryotic and eukaryotic β-galactosidases, and no previous report on β-galactosidase activity of the GH125 α1-6-mannosidase family was found in the literature; the demonstrated substrate structure-dependent dual glycosidase activity thus suggests a new type of bifunctional bacterial enzyme that requires further understanding.

Examining the structural requirements of branched biantennary glycans revealed a slight selectivity toward cleaving the Gal-β1-4GlcNAc residue of the α6-antenna *versus* the α3-antenna. In contrast, the commonly used *Escherichia coli* expressed LacZ enzyme preferentially cleaves the Gal-β1-4GlcNAc residue on the α3-antenna (28, 29) and also produces rearranged allolactose byproducts (34) that can be difficult to remove during purification. Furthermore, our observation that digalactosylated branched glycans have higher reactivity toward α1-6-mannosidase compared to monogalactosylated species to favor the formation of branched monogalactosylated glycans compared to agalactosylated glycans is in contrast to LacZ cleavage, which results in faster formation of agalactosylated glycans. Together, our results suggest that XmGH125 α1-6-mannosidase may have synthetic value to produce specific monogalactosylated species of branched biantennary glycans.

Docking simulations and structural modeling of XmGH125 α1-6-mannosidase provide a potential explanation to its promiscuous catalytic activity and the ability to cleave both Man-α1-6-Man and Gal-β1-4-GlcNAc ligands. Postenergy minimization docking simulations reveal a distinct, shallow, binding pocket on the surface of XmGH125 α1-6-mannosidase when compared to the reference mannosidase 3QRY, which contains a narrow and deep pocket. We assign the naming convention ‘loop-in’ and ‘extended arginine arm’ to describe the binding pocket of XmGH125. In this model, the R459 is extended to form a hydrogen bond to stabilize its interactions with the Gal-β1-4-GlcNAc glycosidic oxygen. Furthermore, the M407 on the disordered loop, adjacent to the ligand is extended and occupies the binding pocket. In contrast, the reference 3QRY mannosidase-binding pocket exhibits a ‘loop-out’ and ‘collapsed arginine arm’ conformation in a deep and likely specific pocket for the Man-α1-6-Man ligand to enter. This deeper pocket of the reference 3QRY mannosidase may be more specific for the unbranched Man-α1-6-Man-containing polysaccharides and may not support the entrance and recognition of branched polysaccharide ligands. A shallow pocket, like XmGH125 α1-6-mannosidase, may limit the energy of interaction with our ligand, therefore promoting a higher rate of cleavage with less specificity for a single glycan substrate and increased overall catalytic turnover. Moreover, this shallow binding pocket may be a critical reason why XmGH125 α1-6-mannosidase is capable of binding branched Gal-β1-4-GlcNAc and unbranched Man-α1-6-Man substrates.

A potential mechanism for which the reference mannosidase (3QRY) cleaves the Man-α1-6-Man substrate was previously identified using X-ray crystallography (31). The authors propose D218 and E393 of the reference mannosidase acts as catalytic acid and base/indirect nucleophile, respectively. Furthermore, this group proposes that E393, together with R405, binds to a water molecule that subsequently acts as the nucleophile toward the anomeric carbon of the terminal mannose to cleave the Man-α1-6-Man disaccharide. Relating that to our sequence, this would be equivalent to E447 and R459, respectively. Although E393 and E447 are similarly positioned following post alignment and superposition because our docking simulations do not use water solvation, it is difficult to identify if a nucleophilic water is used by XmGH125 to perform catalysis. Seeing that R459 also plays an important role in the stabilization of both the native Man-α1-6-Man and unexpected Gal-β-1,4-GlcNAc disaccharide ligands in XmGH125, the potential role of nucleophilic water should be investigated in future structural characterization.

The role of the catalytic acid D218 in the crystal structure of the reference mannosidase (SpGH125, PDB: 3QRY) provides insight into the role of the equivalent aligned residue of XmGH125 α1-6-mannosidase, D274. In the Man-α1-6-Man predicted binding mode, D274 is positioned close to the glycosidic oxygen and C1 of the terminal mannose (5.42 Å), though slightly farther than the 4.7 Å distance reported between the glycosidic oxygen and D218 (D274 equivalent) by Gregg *et al.* (31). Previous modeling of the native α-mannoside ligand within the active site of another similar GH125 mannosidase (CpGH125, PDB: 2NVP) showed that α-mannoside can adopt an energetically allowed ^O^S_2_ pseudo-diequatorial conformation at the C1/C2 hydroxyl groups (*i.e.*, instead of diaxial hydroxyl groups in the ground state). With the anomeric glycosidic bond in a pseudoequatorial position, it can then undergo acid-base/indirect nucleophilic hydrolysis by D220 (D274 equivalent in XmGH125)–E393 (E447 equivalent in XmGH125), respectively (35). Our ligand-protein docking with β-D-galactoside (*i.e.*, the enantiomer used in this study, Fig. 6) supports the requirement of an anomeric glycosidic group in an equatorial position, as this is present in β-D-galactose and is on a similar plane as that of the ^O^S_2_ mannose conformation. However, any stabilization interactions from the pseudoequatorial C2-hydroxyl group of the ^O^S_2_ α-mannoside may not be relevant to β-D-galactose, which has its equatorial C2-OH in an enantiomeric configuration (*i.e.*, on the opposite side of C1). While potential C2-OH interactions would be more applicable to β-L-galactosides, these were not used in this study.

Unfortunately, we were unable to determine how the structure of the overall glycan influences β1-4-galactosidase activity of this α1-6-mannosidase enzyme. Rather, our docking simulation studies only provide a potential rationale how this unexpected hydrolytic activity may be feasible. A potential cause for cleavage of branched, but not unbranched, Gal-β1-4-GlcNAc-containing glycans may be that the monomannose at the branching position of the (pseudo)hybrid and complex *N*-glycans may form other stabilizing interactions with the enzyme to facilitate binding of the Gal-β-1-4-GlcNAc residue into the shallow active site and its subsequent cleavage. A similar phenomenon has been reported with Golgi α-mannosidase II (GMII) of the GH38 family, where α-mannosidase activity is enabled by the presence of a nonreducing GlcNAc residue on the opposite glycan antenna of the glycan substrate that binds to an anchor site away from the catalytic site (36, 37). In the absence of the anchoring GlcNAc residue, an 80-fold reduction in mannosidase activity was observed, whereby the authors suggest residues within the anchor site may provide important glycan stabilization *via* aromatic (Tyr-267), hydrophobic (Pro-298, Trp-299), and hydrogen bonding (His 273) interactions. It was further surprising from our data that the presence of a second nonreducing galactose residue in complex N-glycans 10 and 11 drastically increased galactosidase activity (60%–80% in digalactosylated *versus* ∼10%–15% in monogalactosylated glycans). We hypothesize this may be due to multivalent effects caused by the two nonreducing galactose residues that increase its local concentration and affinity (38). Future work examining how branched glycans facilitate this unexpected β1-4-galactosidase activity warrants further study and is beyond the scope of this article. Our docking simulation methodology has been shown to provide an accurate estimation of ligand–protein interaction (39), but it is important to state that docking simulations do not necessarily capture what is occurring in a biological state. Because of present computational limitations and lack of a crystal structure for apo- and holo-XmGH125, future work aims to structurally investigate the dual catalytic activity phenomena in greater detail.

While our data demonstrate a decrease in ligand epitope specificity of α1-6-mannosidase from *X. manihotis*, it does not take away from the usefulness of this enzyme when used in the proper context. For example, it is still useful in mapping mannose-α1-6-mannose linkages in agalactosylated high mannose structures. However, their applications in complex and unknown biological samples should be used with further controls to ensure proper data interpretation and use multiple methods to validate proposed glycan structures.

## Experimental procedures

### Synthesis of truncated monogalactosylated and digalactosylated glycans

Sialoglycoprotein (SGP) was extracted from commercial egg yolk as previously described (9). Briefly, egg yolk isolated from 20 eggs using first washed with diethyl ether (400 ml × 3) by vigorous stirring and filtration. SGP was extracted from the resulting solid using 40% acetone (400 ml × 2, stirring overnight). The supernatant was filtered, washed with 200 ml of 40% acetone, evaporated, and concentrated to afford an off-white solid. The solid was resuspended in water, passed through a Buchner funnel containing 15 g activated carbon, and washed with 2.5% acetonitrile. SGP was eluted with 25% acetonitrile containing 0.1% TFA. The eluted sialoglycans were evaporated and lyophilized. Glycans were cleaved from SGP (8.0 mg diluted in 1.0 ml PBS) between the two core GlcNAc residues using Endo S containing a chitin-binding domain (New England Biolabs, catalog no.: #P0741S) that was immobilized onto chitin resin (New England Biolabs, catalog no.: #S6651S, 10 μl Endo S was premixed with 1.0 ml of chitin resin slurry overnight at 4 °C). Glycans were simultaneously desialylated using 5 μl of α2-3,6,8 neuraminidase (New England Biolabs, catalog no.: #P0720S) upon incubation overnight at 37 °C. The reaction progress was monitored by HPAEC-PAD. Following completion of the reaction, Endo S resin was removed by filtration, and the reaction was desalted by passing it through a solid phase extraction tube containing activated carbon. Salts were removed by passing through 2.5% acetonitrile in Milli-Q (MQ) water, and glycans were eluted with 25% acetonitrile, collected, evaporated, and lyophilized to dryness. The resulting glycan mixture comprising monogalactosylated and digalactosylated glycans (40) was resuspended at 5 mg/ml with MQ water. To 0.5 ml (2.5 mg) of this mixture, 75 μl of KCl (500 mM), 75 μl of a buffer containing 500 mM sodium acetate and 50 mM CaCl_2_ (pH 5.7), and 75 μl of LacZ β1-4-galactosidase (Roche, catalog no.: #10105031001) was added and the reaction was incubated at 37 °C for 6 h. The reaction was monitored by HPAEC-PAD until the desired amounts of truncated monogalactosylated glycans appeared. The reaction was then combined with the initial mixture comprising monogalactosylated and digalactosylated glycans and lyophilized. The solution was then resuspended to 2.5 mg/ml in MQ water, filtered through a 0.2 μm nylon filter, and purified using HPLC equipped with a HyperCarb PGC column (Thermo Fisher; catalog no.: #35005-154630, 4.6 cm × 150 cm, 5 μm particle size) and a gradient of 4.0% to 13.7% MP-A:MP-B (MP-A: 95% acetonitrile in MQ H_2_O containing 0.1% TFA; MP-B: MQ H_2_O containing 0.1% TFA) at 2 ml/min over 41 min at 40 °C. Glycans were detected at 214 nm using a UV detector and collected using a fraction collector. The β- and α-anomers of the truncated glycans eluted at the following approximate retention times: monogalactosylated glycan 7 (α3 antenna), 27.7 min and 31.5 min; monogalactosylated glycan 9 (α6 antenna), 29.6 min and 33.7 min; digalactosylated glycan 10, 34.8 min and 39.0 min.

Purity of all collected fractions of glycans was confirmed by HPAEC-PAD analysis and mass spectrometry. Impure fractions were combined and repurified using the same aforementioned HPLC method, while pure samples were combined, lyophilized, and stored at −80 °C until use.

### Synthesis of (pseudo)hybrid and linear galactosylated glycans

Purified monogalactosylated complex truncated glycans 7 and 9 were first treated with β-*N*-acetylglucosaminidase (New England Biolabs, catalog no.: #P0744) to cleave the nonreducing terminal GlcNAc residue of glycans 1 and 2. Reactions were monitored by HPAEC-PAD, and glycans were purified using HPLC as described previously with the following gradient: 4.0% to 12.0% MP-A:MP-B (MP-A: 95% acetonitrile in MQ H_2_O containing 0.1% TFA; MP-B: MQ H_2_O containing 0.1% TFA) at 2 ml/min over 34 min, followed by an increase 50% MP-A for the next 1.0 min, then maintained at 50% MP-A for another 3 min, at 40 °C. As aforementioned, purified fractions were confirmed by HPAEC-PAD, combined, and lyophilized. To pseudohybrid glycan 1, α1-2,3-mannosidase (New England Biolabs, catalog no.: #P0729) was added to cleave the branching monomannose residue on the α3 antenna to form linear pentasaccharide 5. The resulting product was then purified by HPLC using the same method. The β- and α-anomers of glycan retention times are as follows: pseudohybrid glycan 1, 38.8 min and 38.9 min; hybrid glycan 2, 26.8 min and 31.5 min; linear glycan 5, 28.7 min and 33.6 min.

### Isolation of G2 glycan with chitobiose core at the reducing end (GlcNAc-GlcNAc), and synthesis of Man-α1-6-Man-GlcNAc2 glycan

G2 glycan 11 was isolated from SGP in a similar method as aforementioned. PNGaseF was used instead of Endo S resin to obtain the glycan with the full chitobiose core at the reducing end (GlcNAc-GlcNAc). G2 11 was then treated with Lac Z β1-4-galactosidase and purified by PGC-HPLC as aforementioned to obtain the glycan 13 bearing a monogalactosylated at the α6 antenna. Glycan 13 was diluted to 1 mg/ml (190 μl) with MQ water and 10 μl was GlycoBuffer 1 (10×) was added. About 0.5 μl of β-*N*-acetylglucosaminidase was added, and the reaction was incubated to completion for 45 min. About 4.8 μl of α1-2,3 mannosidase and 27.5 μl LacZ β1-4-galactosidase were then added and the reaction was incubated for 2 days at 37 °C. Reaction completion was monitored by HPAEC-PAD, and purification was performed using HC-HPLC and the aforementioned method. The β- and α-anomers of Man-α1-6-Man-GlcNAc2 eluted at 29 and 37.5 min.

### Enzymatic deglycosylation methods using α1-6-mannosidase

For activity studies, all glycans were determined using HPAEC-PAD and diluted to achieve the same glycan concentrations for all reactions. Final reaction concentrations are stated in each Figure. As a representative example for a 10 μl reaction with 100 μM glycan and 2 U/μl of α1-6-mannosidase enzyme, 8.5 μl of 120 μM glycans (previously diluted in MQ H_2_O), 1.0 μl of 10× GlycoBuffer 1 (New England Biolabs, catalog no.: #B1727S), and 0.5 μl of α1-6-mannosidase from *X. manihotis* (New England Biolabs P0727S, 40,000 U/ml) were added and the reaction was incubated at 37 °C for the stated amount of time. Multiple lots purchased at different times were used for activity studies. For reactions with mannosidase inhibitor dMNJ, 25 μM of glycans were used with an enzyme concentration of 0.05 U/μl (for Man-α1-6-Man-GlcNAc_2_ glycan) or 2.0 U/μl (for G2T glycan 10) and an inhibitor/enzyme ratio of 10 or 100 nmol_dMNJ_/U_α1-6-mannosidase_. As a representative example for a 9 μl reaction of 25 μM Man-α1-6-Man-GlcNAc_2_ with 0.05 U/μl α1-6-mannosidase and 5 mM dMNJ (*i.e.*, 100 nmol_dMNJ_/U_α1-6-mannosidase_), 0.9 μl of α1-6-mannosidase (diluted to 0.5 U/μl in 1× GlycoBuffer 1) was added to a solution of dMNJ (6.85 μl, 6.57 mM) and 0.7 μl GlycoBuffer 1 (10×), which was then incubated for 15 min at 37 °C. About 0.56 μl of Man-α1-6-Man-GlcNAc2 (401.5 μM) was added and the reaction was further incubated for the desired amount of time. Reactions were stopped by diluting with MQ water and heating to 70 °C for 15 min, which were then monitored using HPAEC-PAD. One unit is defined by the manufacturer as the amount of α1-6-mannosidase that is needed to hydrolyze greater than 95% of the nonreducing α-D-mannose residue from 1 nmol of the trisaccharide Man-α1-6-Man-α1-6-Man-7-amino-4-methyl-coumarin in a total volume of 10 μl, after incubation at 37 °C for 1 h.

### HPAEC-PAD analysis of the glycan reactions with glycosidases

HPAEC-PAD analyses were performed on Dionex IC-3000 or IC-6000 instruments, using a Dionex CarboPac PA200 IC column (3 mm × 250 mm, 5.5 μm particle size, catalog no.: #062896) and a Gold Standard PAD waveform with an AgCl electrode. For monitoring glycosidase reactions in the synthesis of glycans 7 to 10 and α1-6-mannosidase concentration scan with G2T glycan 10, a flow rate of 0.4 ml/min and a gradient of 20%/0%/80% to 60%/20%/20% of MP-A/MP-B/MP-C (MP-A: 200 mM NaOH; MP-B: 150 mM sodium acetate in 200 mM NaOH; MP-C: MQ H_2_O) over 10 min at 30 °C was used to monitor glycan products.

For monitoring other glycan substrate reactions with *X. manihotis* α1-6-mannosidase and Lac Z, the following HPAEC gradient was used: 20%/0%/80% to 25%/5%/70% of MP-A/MP-B/MP-C over 25 min at 30 °C, followed by an increase to 0/80/20 over the next 3 min, and then re-equilibration to 20/0/80 over the next 10 min. A flow rate of 0.4 ml/min was used for all injections.

### PGC LC-MS/MS analyses of proteolytic products of glycan substrates by glycosidases

Analyses of glycan products were carried out on an Orbitrap Fusion mass spectrometer (Thermo Fisher Scientific) coupled with an Acquity ultraperformance UPLC M-class system (Waters). Following the digestion of glycans by mannosidase α1-2,3 mannosidase1-6 and β1-4-galactosidase, the glycan products were desalted using a PGC microcolumn and freeze-dried by SpeedVac. Purified samples were dissolved in water and loaded into an in-house packed PGC trap column (150 μm × 2 cm, 3 Å particle size) and subsequently separated by an analytical PGC column (100 μm × 25 cm, 3 Å particle size) as described previously (26). Glycans were trapped for 3 min at the flow rate of 5 μl/min and eluted at 600 nl/min using a LC gradient run of 10% to 25% acetonitrile in 0.1% formic acid (FA) at a duration of 38 min. The MS measurements were conducted on the Orbitrap at a resolution of 120,000, and MS/MS fragmentation of multiply charged ions was performed on the ion-trap using collision-induced dissociation at the normalized collision energy of 25%. The glycans were manually identified by data interpretation of accurate masses of glycans in the MS spectra and MS/MS fragmentations.

### Reversed phase LC-MS/MS identification of XmGH125 α1-6-mannosidase

XmGH125 α1-6-mannosidase was reduced with 10 mM DTT, alkylated with 55 mM iodoacetamide, dialyzed against 10 mM ammonium bicarbonate, and dried using a CentriVap centrifugal concentrator (Labconco). The protein was subsequently digested at 37 °C overnight using either sequencing grade trypsin (Promega) or chymotrypsin from bovine pancreas (Roche Diagnostics GmbH) in 25 mM ammonium bicarbonate at 1:100 ratio of enzyme to protein substrates. The resulting peptides were dried and reconstituted with 0.2% FA and identified by LC-MS/MS. The peptides were trapped by a NanoEase m/z symmetry C18 trap column (100 Å, 5 μm, 180 μm I.D. × 20 mm length) for 3 min at the flow rate of 5 ml/min using solvent A (0.1% FA) at 300 μl/min and separated on a NanoEase m/z HSS C18 T3 analytical column (100 Å, 1.8 μm, 75 μm I.D. × 150 mm length, Waters) at the flow rate of 300 nl/min for 90 min. A linear gradient from 2% to 30% of solvent B (0.1% FA in 99.9% acetonitrile, Waters) at the duration of 65 min was used for peptide elution, followed by flushing with 85% solvent B for 10 min and re-equilibrating the column with solvent A for 15 min. MS survey scan was acquired with a high resolution of 120,000 at the mass region of m/z 350 to 1800, and MS/MS measurements were performed on peptides at multicharged ions of 2+ to 7+ using collision-induced dissociation at the normalized collision energy of 28%. Dynamic exclusion was set to 30 s.

The raw LC-MS/MS data were searched against the protein sequences of *X. manihotis* GHs downloaded from the NCBI database (https://www.ncbi.nlm.nih.gov/) using Mascot Server (version 2.6.0; Matrix Science). The search parameters were restricted to tryptic or chymotryptic peptides at a maximum of two missed cleavages. Cys carbamidomethylation was designated as a fixed modification. Oxidation of methionine and deamidation of Asn and Gln were considered as variable modifications. Mass tolerances were set up to 10 ppm for Orbitrap MS ions and 0.8 Da for ion-trap MS/MS fragment ions. Peptide assignments were filtered by the significance threshold *p* value <0.05, and the identified sequences were verified manually based on MS/MS spectra.

### Enzyme model generation

The XmGH125 α1-6-mannosidase sequence (WP_017155573) was obtained from the NCBI database, and a structural model was generated with the Phyre2 server (http://www.sbg.bio.ic.ac.uk/phyre2/), an online protein structure prediction tool (41). α1-6-mannosidase (PDB: 3QRY) and *E. coli* Lac Z β1-4-galactosidase (PDB: 1DP0) were obtained from the RCSB PDB to be used as positive controls in the docking simulations (31, 42, 43). Ligand structure files were created using ChemDraw software (PerkinElmer 2021). Protein models and ligands underwent a ‘QuickPrep’ protocol in MOE 2020 (Chemical Computing Group 2020). The ‘QuickPrep’ tool corrects detailed structural errors in the molecules such as incorrect charges, removing selenium atoms, ambiguous sequence identities, etc. The molecule is then protonated to the correct protonation state and hydrogens are added. The structures were energy minimized using an Amber10:EHT force field to an RMS gradient of 0.1 kcal/mol/Å (44).

### Protein–protein alignment and superposition

The multiple sequence alignment of the XmGH125 α1-6-mannosidase sequences with other homology GH125 α1-6-mannosidases downloaded from the UniProKB database was generated by the Clustal Omega program (https://www.ebi.ac.uk/Tools/msa/clustalo/). Protein sequences were also aligned using BLAST, to identify proteins of high sequence similarity (45). The XmGH125 sequence was inputted into the MOE ‘PDB Search’ algorithm to query the XmGH125 sequence against all structurally determined PDB entities. Proteins were aligned in MOE using a BLOSUM62 scoring matrix and structurally superimposed using the alignment results (46).

### Protein-ligand docking

Polysaccharides and trimmed disaccharide sugars were docked into the XmGH125 α1-6-mannosidase and positive control structures (PDBs: 3QRY, 1DP0) in MOE using a general docking algorithm. Triangle Matcher placement with London dG scoring and Rigid Receptor refinement with GBVI/WSA dG scoring were used (39). London dG and GBVI/WSA dG scoring functions estimate the free binding energy of a ligand in a given pose; however, GBVI/WSA dG scoring is used for refinement as it takes into account force field, charge, surface area exposure, and Van der Waals contribution (39). Final docking poses were ranked by free energy score and visually inspected by the user.

## Data availability

Data generated or analyzed during this study are included in this article and Supporting Information files.

## Supporting information

This article contains supporting information.

## Conflict of interest

The authors declare that they have no conflicts of interest with the contents of this article.

## References

[1] Lombard V., Golaconda Ramulu H., Drula E., Coutinho P.M., Henrissat B. (2014). The carbohydrate-active enzymes database (CAZy) in 2013. Nucleic Acids Res..

[2] Ladeveze S., Laville E., Despres J., Mosoni P., Potocki-Veronese G. (2017). Mannoside recognition and degradation by bacteria. Biol. Rev. Camb. Philos. Soc..

[3] Dupoiron S., Zischek C., Ligat L., Carbonne J., Boulanger A., Duge de Bernonville T. (2015). The N-glycan cluster from Xanthomonas campestris pv. campestris: a toolbox for sequential plant N-glycan processing. J. Biol. Chem..

[4] Lederkremer G.Z. (2009). Glycoprotein folding, quality control and ER-associated degradation. Curr. Opin. Struct. Biol..

[5] Parodi A.J. (2000). Protein glucosylation and its role in protein folding. Annu. Rev. Biochem..

[6] Frappaolo A., Karimpour-Ghahnavieh A., Sechi S., Giansanti M.G. (2020). The close relationship between the Golgi trafficking machinery and protein glycosylation. Cells.

[7] Mathew C., Weiss R.G., Giese C., Lin C.W., Losfeld M.E., Glockshuber R. (2021). Glycan-protein interactions determine kinetics of N-glycan remodeling. RSC Chem. Biol..

[8] Klamer Z., Hsueh P., Ayala-Talavera D., Haab B. (2019). Deciphering protein glycosylation by computational integration of on-chip profiling, glycan-array data, and mass spectrometry. Mol. Cell. Proteomics.

[9] Sun B., Bao W., Tian X., Li M., Liu H., Dong J. (2014). A simplified procedure for gram-scale production of sialylglycopeptide (SGP) from egg yolks and subsequent semi-synthesis of Man3GlcNAc oxazoline. Carbohydr. Res..

[10] Koizumi A., Matsuo I., Takatani M., Seko A., Hachisu M., Takeda Y. (2013). Top-down chemoenzymatic approach to a high-mannose-type glycan library: synthesis of a common precursor and its enzymatic trimming. Angew. Chem. Int. Ed. Engl..

[11] Chao Q., Ding Y., Chen Z.H., Xiang M.H., Wang N., Gao X.D. (2020). Recent progress in chemo-enzymatic methods for the synthesis of N-glycans. Front. Chem..

[12] Toonstra C., Wu L., Li C., Wang D., Wang L.X. (2018). Top-down chemoenzymatic approach to synthesizing diverse high-mannose N-glycans and related neoglycoproteins for carbohydrate microarray analysis. Bioconjug. Chem..

[13] Goetze A.M., Liu Y.D., Zhang Z., Shah B., Lee E., Bondarenko P.V. (2011). High-mannose glycans on the Fc region of therapeutic IgG antibodies increase serum clearance in humans. Glycobiology.

[14] Coelho V., Krysov S., Ghaemmaghami A.M., Emara M., Potter K.N., Johnson P. (2010). Glycosylation of surface Ig creates a functional bridge between human follicular lymphoma and microenvironmental lectins. Proc. Natl. Acad. Sci. U. S. A..

[15] Lalonde M.E., Durocher Y. (2017). Therapeutic glycoprotein production in mammalian cells. J. Biotechnol..

[16] Hollister J., Grabenhorst E., Nimtz M., Conradt H., Jarvis D.L. (2002). Engineering the protein N-glycosylation pathway in insect cells for production of biantennary, complex N-glycans. Biochemistry.

[17] Ward B.J., Landry N., Trepanier S., Mercier G., Dargis M., Couture M. (2014). Human antibody response to N-glycans present on plant-made influenza virus-like particle (VLP) vaccines. Vaccine.

[18] Bardor M., Faveeuw C., Fitchette A.C., Gilbert D., Galas L., Trottein F. (2003). Immunoreactivity in mammals of two typical plant glyco-epitopes, core alpha(1,3)-fucose and core xylose. Glycobiology.

[19] Palacpac N.Q., Yoshida S., Sakai H., Kimura Y., Fujiyama K., Yoshida T. (1999). Stable expression of human beta1,4-galactosyltransferase in plant cells modifies N-linked glycosylation patterns. Proc. Natl. Acad. Sci. U. S. A..

[20] Wang T., Voglmeir J. (2014). PNGases as valuable tools in glycoprotein analysis. Protein Pept. Lett..

[21] Wong-Madden S.T., Landry D. (1995). Purification and characterization of novel glycosidases from the bacterial genus Xanthomonas. Glycobiology.

[22] Athanasopoulos V.I., Niranjan K., Rastall R.A. (2004). Regioselective synthesis of mannobiose and mannotriose by reverse hydrolysis using a novel 1,6-α-d-mannosidase from Aspergillus phoenicis. J. Mol. Catal. B Enzym..

[23] Wang L.T., Lin M.H., Liu K.Y., Chiou S.S., Wang S.N., Chai C.Y. (2021). WLS/wntless is essential in controlling dendritic cell homeostasis via a WNT signaling-independent mechanism. Autophagy.

[24] Hamilton B.S., Wilson J.D., Shumakovich M.A., Fisher A.C., Brooks J.C., Pontes A. (2017). A library of chemically defined human N-glycans synthesized from microbial oligosaccharide precursors. Sci. Rep..

[25] Schiller B., Hykollari A., Yan S., Paschinger K., Wilson I.B. (2012). Complicated N-linked glycans in simple organisms. Biol. Chem..

[26] She Y.M., Tam R.Y., Li X., Rosu-Myles M., Sauve S. (2020). Resolving isomeric structures of native glycans by nanoflow porous graphitized carbon chromatography-mass spectrometry. Anal. Chem..

[27] Song T., Ozcan S., Becker A., Lebrilla C.B. (2014). In-depth method for the characterization of glycosylation in manufactured recombinant monoclonal antibody drugs. Anal. Chem..

[28] van den Eijnden D.H., Blanken W.M., van Vliet A. (1986). Branch specificity of β-d-galactosidase from Escherichia coli. Carbohydr. Res..

[29] Calderon A.D., Zhou J., Guan W., Wu Z., Guo Y., Bai J. (2017). An enzymatic strategy to asymmetrically branched N-glycans. Org. Biomol. Chem..

[30] Kato A., Kato N., Kano E., Adachi I., Ikeda K., Yu L. (2005). Biological properties of D- and L-1-deoxyazasugars. J. Med. Chem..

[31] Gregg K.J., Zandberg W.F., Hehemann J.H., Whitworth G.E., Deng L., Vocadlo D.J. (2011). Analysis of a new family of widely distributed metal-independent alpha-mannosidases provides unique insight into the processing of N-linked glycans. J. Biol. Chem..

[32] Bendtsen J.D., Nielsen H., Widdick D., Palmer T., Brunak S. (2005). Prediction of twin-arginine signal peptides. BMC Bioinformatics.

[33] Bazzoli F., Fossi S., Sottili S., Pozzato P., Zagari R.M., Morelli M.C. (1995). The risk of adenomatous polyps in asymptomatic first-degree relatives of persons with colon cancer. Gastroenterology.

[34] Juers D.H., Matthews B.W., Huber R.E. (2012). LacZ beta-galactosidase: structure and function of an enzyme of historical and molecular biological importance. Protein Sci..

[35] Alonso-Gil S., Males A., Fernandes P.Z., Williams S.J., Davies G.J., Rovira C. (2017). Computational design of experiment unveils the conformational reaction coordinate of GH125 alpha-mannosidases. J. Am. Chem. Soc..

[36] Shah N., Kuntz D.A., Rose D.R. (2008). Golgi alpha-mannosidase II cleaves two sugars sequentially in the same catalytic site. Proc. Natl. Acad. Sci. U. S. A..

[37] Zhong W., Kuntz D.A., Ember B., Singh H., Moremen K.W., Rose D.R. (2008). Probing the substrate specificity of Golgi alpha-mannosidase II by use of synthetic oligosaccharides and a catalytic nucleophile mutant. J. Am. Chem. Soc..

[38] van Heteren J., Pieters R.J., Tiwari V.K. (2020). Carbohydrates in Drug Discovery and Development.

[39] Corbeil C.R., Williams C.I., Labute P. (2012). Variability in docking success rates due to dataset preparation. J. Comput. Aided Mol. Des..

[40] Liu L., Prudden A.R., Bosman G.P., Boons G.J. (2017). Improved isolation and characterization procedure of sialylglycopeptide from egg yolk powder. Carbohydr. Res..

[41] Kelley L.A., Mezulis S., Yates C.M., Wass M.N., Sternberg M.J. (2015). The Phyre2 web portal for protein modeling, prediction and analysis. Nat. Protoc..

[42] Berman H.M., Westbrook J., Feng Z., Gilliland G., Bhat T.N., Weissig H. (2000). The Protein Data Bank. Nucleic Acids Res..

[43] Juers D.H., Jacobson R.H., Wigley D., Zhang X.J., Huber R.E., Tronrud D.E. (2000). High resolution refinement of beta-galactosidase in a new crystal form reveals multiple metal-binding sites and provides a structural basis for alpha-complementation. Protein Sci..

[44] Ponder J.W., Case D.A. (2003). Force fields for protein simulations. Adv. Protein Chem..

[45] Altschul S.F., Gish W., Miller W., Myers E.W., Lipman D.J. (1990). Basic local alignment search tool. J. Mol. Biol..

[46] Henikoff S., Henikoff J.G. (1992). Amino acid substitution matrices from protein blocks. Proc. Natl. Acad. Sci. U. S. A..

