# Supplementary material for: Unusual β1-4-galactosidase activity of an α1-6-mannosidase from Xanthomonas manihotis in the processing of branched hybrid and complex glycans
Source: J Biol Chem. 2022 Jul 31;298(9):102313. doi: 10.1016/j.jbc.2022.102313 (PMC9425025; doi:10.1016/j.jbc.2022.102313)
Supplement: Supplemental Figures S1–S10 and Table S1 [file mmc1.pdf]

Supplementary Materials

Unusual β1-4 galactosidase activity of an α1-6 mannosidase from *Xanthomonas manihotis* in the processing of branched hybrid and complex glycans

Yi-Min She,[1] Kody Klupt,[2] Grayson Hatfield, [1] Zongchao Jia,[2] Roger Y. Tam[1]*

1. Centre for Biologics Evaluation, Biologic and Radiopharmaceutical Drugs Directorate, Health Canada, Ottawa, Ontario K1A 0K9, Canada
2. Department of Biomedical and Molecular Sciences, Queen's University, Kingston, Ontario K7L 3N6 Canada

Table of Contents

Table of Contents……………………………………………………………………………………………..Page S1

Figure S1 Mass Spectrometric analyses of the products generated from unexpected β1-4-galactosidase reactivity of *X. manihotis* α1-6-mannosidase with branched monogalactosylated glycans……………………….….Page S2

Figure S2 Expected α1-3 mannosidase reactivity of branched monogalactosylated glycans with *X. manihotis* α1-2,3 mannosidase. ……………..…......................…………....………………………………..................Page S3

Figure S3 SDS-PAGE gel of *X. manihotis* α1-6 mannosidase….…...…………………….………………..…Page S4

Figure S4 α1-6 mannosidase activity is retained in *X. manihotis* α1-6 mannosidase ……..………………..…Page S5

Figure S5 Evaluation of the β1-4 galactosidase activity of *X. manihotis* α1-6 mannosidase on various digalactosylated glycan concentrations……………………………………………………………………………….Page S6

Figure S6 Relative activity of α1,6 mannosidase and β1-4 galactosidase in *X. manihotis* α1-6 mannosidase..Page S7

Figure S7 Inhibition of both α1-6 mannosidase and β1-4 galactosidase activities in *X. manihotis* α1-6 mannosidase by 1-deoxymannojirimycin (dMNJ) …………………………………………………………………Page S8

Figure S8 Structural identification of glycan substrates reacted with *X. manihotis* α1-6 mannosidase under the treatments with and without 1-deoxymannojirimycin (dMNJ)………..…………………………..Page S9

Figure S9 Protein sequence alignment of α1-6 mannosidases in the GH125 family.……..…….….………...Page S10

Figure S10 Protein modelling reveals a shallow pocket of α1-6 mannosidase from *X. manihotis* (XmGH125) promoting ligand-receptor promiscuity.……..….……Page S11

Table S1 LC MS/MS identification of the tryptic *X. manihotis* α1,6 mannosidase….………………..….….…….Page S12

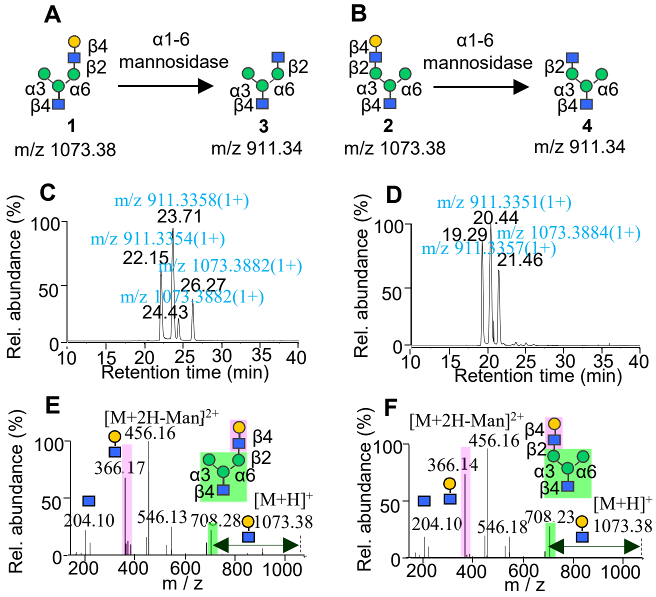

Figure S1. Mass Spectrometric analyses of the products generated from unexpected β1-4-galactosidase reactivity of *X. manihotis α1-6-man*nosidase with branched monogalactosylated glycans. (A, B) Reaction schemes of branched monogalactosylated glycan isomers **1** and **2** (100 µM) with α1-6-mannosidase from *X. manihotis* (6 U/µL, 20 µL, 37 °C) to form unexpected degalactosylated products. (C, D) Total ion chromatograms (TIC) of glycans **1** and **2** following α1-6-mannosidase treatments, analyzed by PGC-LC-MS/MS; the peaks at m/z 1073.39 and m/z 911.34 correspond to the branched monogalactosylated glycan starting materials ($[GalGlcNAcMan_3GlcNAc+H]^+$) and degalactosylated products ($[GlcNAcMan_3GlcNAc+H]^+$), respectively. The observed two peaks with the same m/z ratio in each TIC correspond to β- and α-anomers of each compound that are separated using PGC-LC, and labeled with high abundance singly charged ions even though their doubly charged ions are also observed. (E-F) MS/MS fragmentation of the doubly charged ions of the starting branched monogalactosylated glycans, showing the loss of a branching side chain to form a diagnostic GalGlcNAc disaccharide ion at m/z 366.17 and its complementary fragment $[(M\text{-}GalGlcNAc+H)^+]$ at m/z 708.28. The identification of the degraded products by tandem mass spectrometry is shown in Fig. 1.

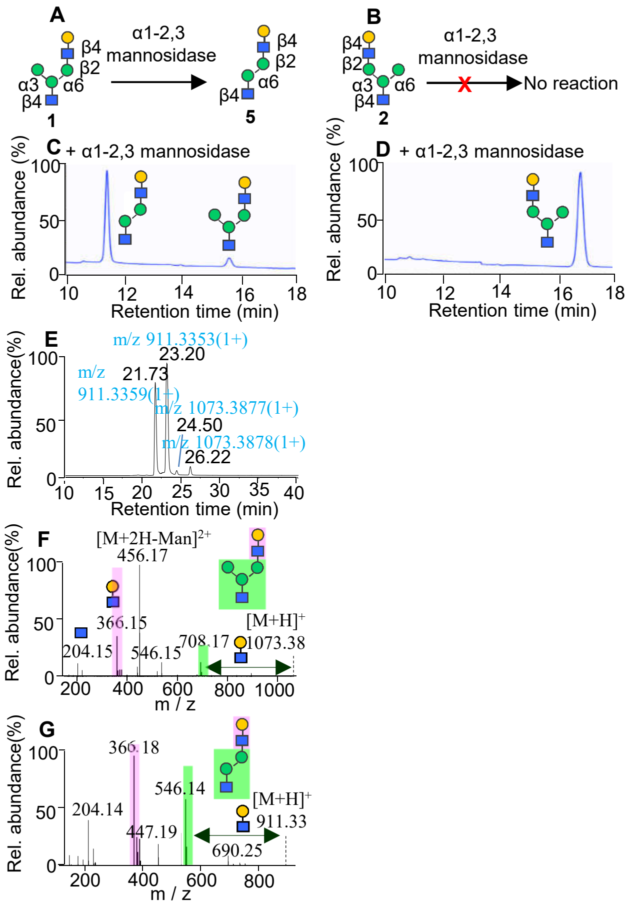

Figure S2. Expected α1-3 mannosidase reactivity of branched monogalactosylated glycans with *X. manihotis α1-2,3* mannosidase. (A, B) Reaction scheme of branched monogalactosylated glycans (**1** and **2**, 100 µM) with α1-2,3-mannosidase (1.6 U / µL, 20 µL / reaction, 37 ^oC). (C, D) HPAEC-PAD chromatograms of the reacted glycan 1 containing a free monomannose at the α3 antenna, and the unreacted glycan **2** containing a branched GalGlcNAc at the α3 antenna. (E) Total ion chromatogram of glycan **1** reacted with α1-2,3-mannosidase, analyzed by PGC-LC-MS/MS. (F) MS/MS spectrum of glycan **1** at m/z 1073.38. (G) MS/MS spectrum of de-mannosylated glycan **5** at m/z 911.33.

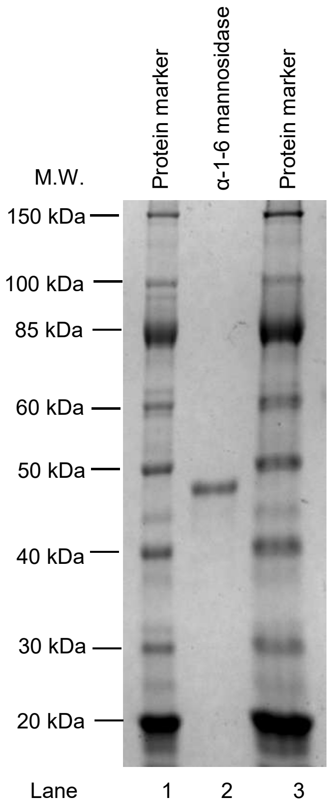

Figure S3. SDS-PAGE gel of *X. manihotis* α1-6 mannosidase. Standard protein marker (Lane 1 and 3), α1-6 mannosidase from *X. manihotis* (Lane 2).

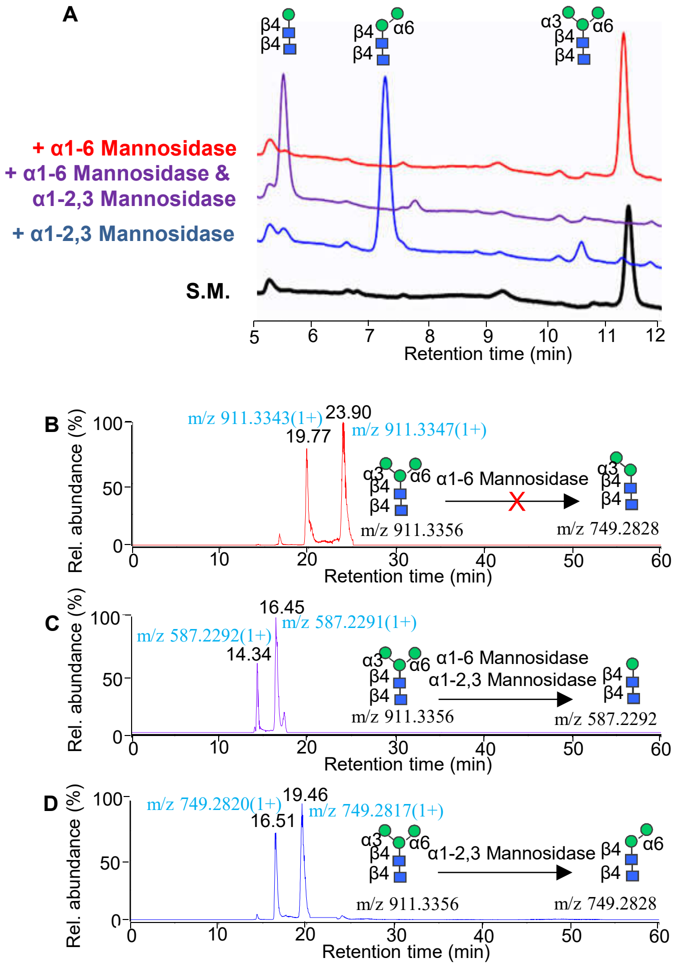

Figure S4. α1-6 mannosidase activity is retained in *X. manihotis* α1-6 mannosidase. (A) HPAEC-PAD chromatograms of paucimannose ($Man_3GlcNAc_2$, black trace) reacted with α1-6 mannosidase alone (red trace), α1-6 mannosidase with α1-2,3 mannosidase (purple trace), or α1-2,3 mannosidase alone (blue trace). (B-D) PGC-LC-MS/MS ion chromatograms of the reaction of paucimannose with glycosidases: (B) α1-6 mannosidase alone, (C) α1-6 mannosidase with α1-2,3 mannosidase, or (D) α1-2,3 mannosidase alone.

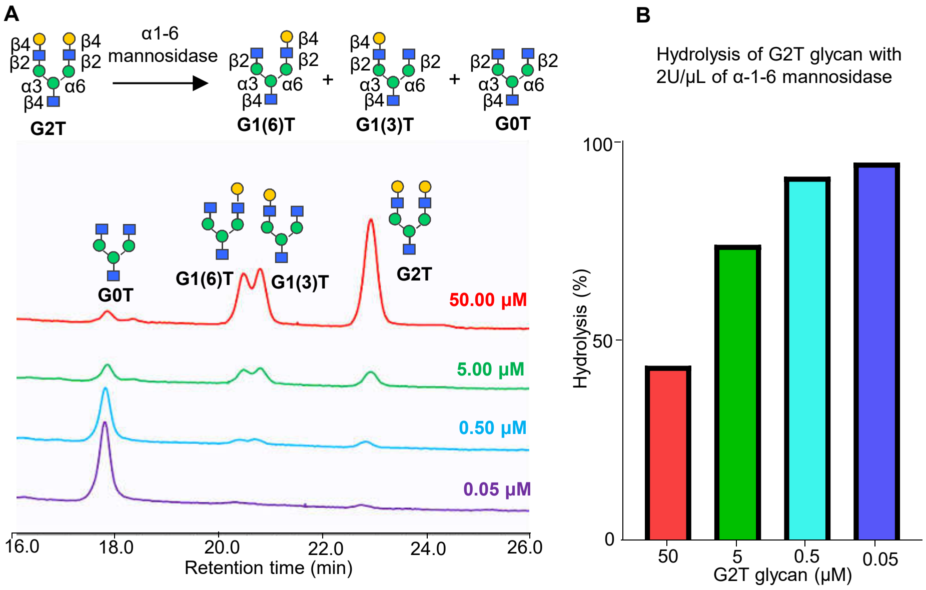

Figure S5. Evaluation of the β1-4 galactosidase activity of *X. manihotis* α1-6 mannosidase on various digalactosylated glycan concentrations. (A) HPAEC-PAD chromatograph traces showing the relative amounts of products generated from the hydrolysis of various concentrations of digalactosylated G2T glycan **10** with 2U / µL of enzyme at 37 °C for 3 hrs. (B) Percentage of the hydrolysis of the various concentrations of digalactosylated G2T glycan substrate.

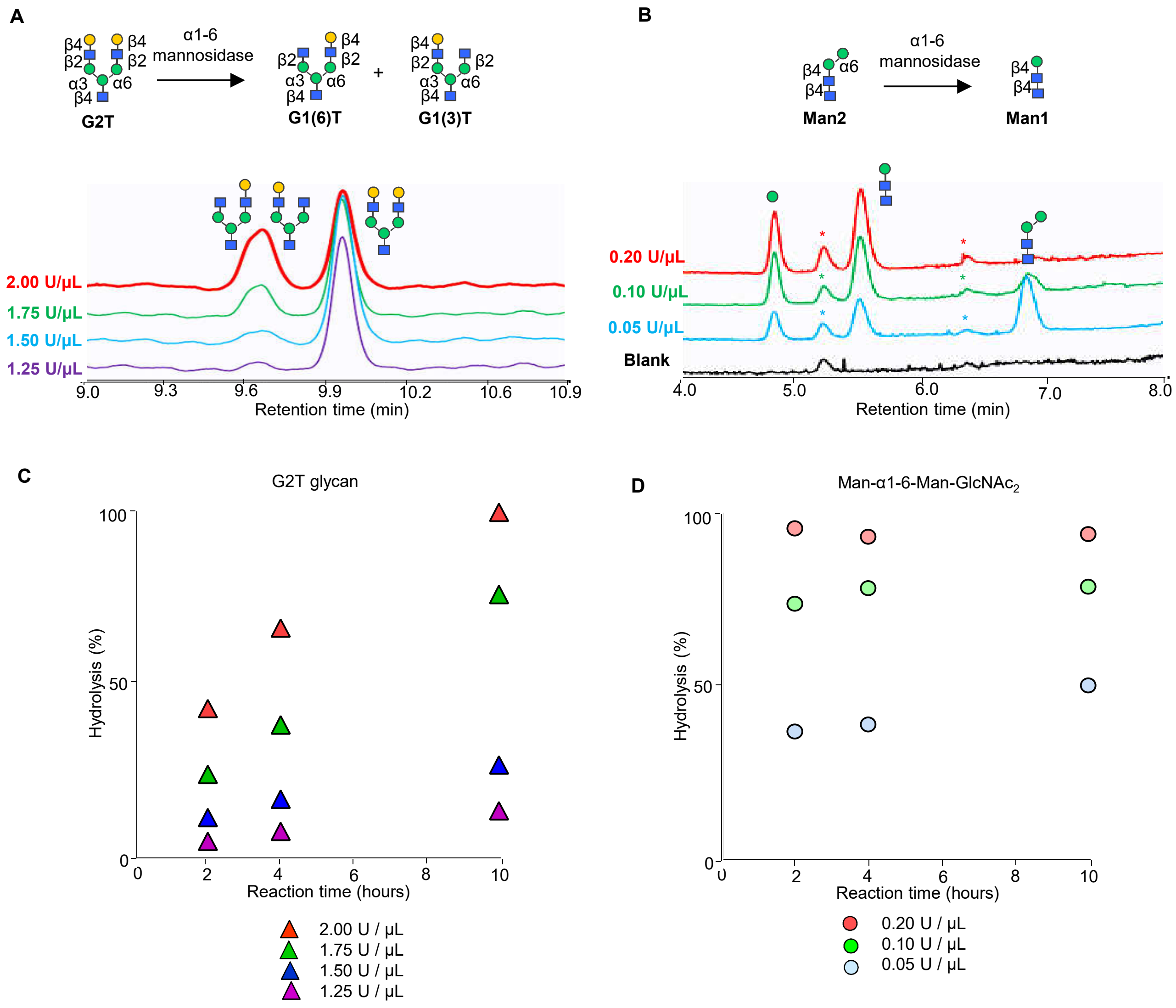

Figure S6. Relative activity of α1-6 mannosidase and β1-4 galactosidase in *X. manihotis* α1-6 mannosidase. (A,B) HPAEC-PAD chromatographs of the products generating from the hydrolysis of digalactosylated G2T glycan **10** and Man-α1-6-Man tetrasaccharide, at various enzyme concentrations at 37 °C for 2 hrs. Background peaks associated with blank sample injections are denoted with asterisks. (C,D) Percentage of the hydrolysis of glycan substrates at time intervals of 2, 4 or 10 hrs at 37 °C upon treatments with varying enzyme concentrations. 25 µM of each glycan substrate was used for each reaction.

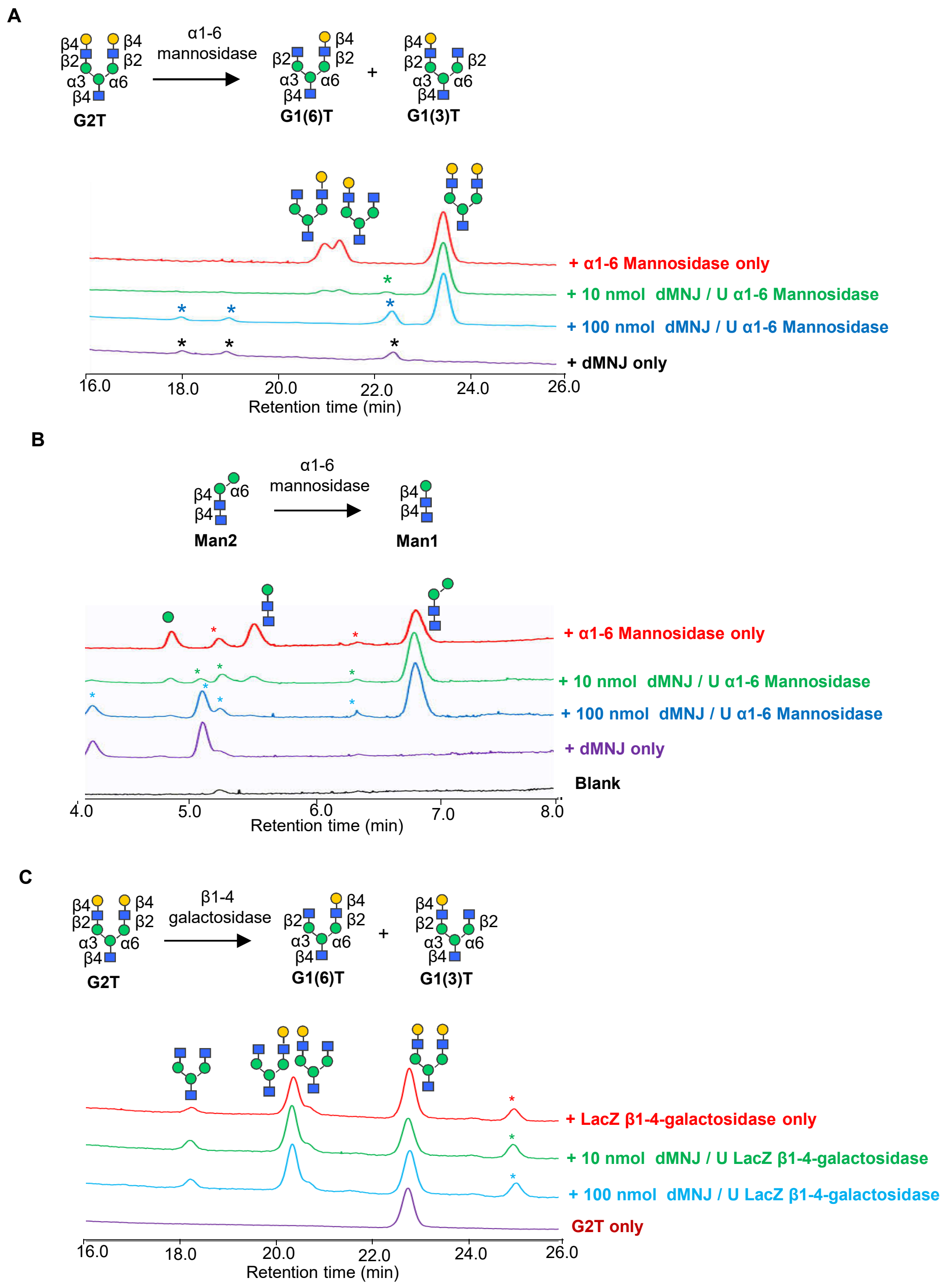

Figure S7. Inhibition of both α1-6 mannosidase and β1-4 galactosidase activities in *X. manihotis* α1-6 mannosidase by 1-deoxymannojirimycin (dMNJ). HPAEC-PAD chromatographs showing the inhibition of hydrolysis of (A) digalactosylated G2T glycan **10**, and (B) Man-α1-6-Man tetrasaccharide, at 10 nmol or 100 nmol of dMNJ per unit of α1-6 mannosidase. At 10 nmol / unit, approximately 75% of enzyme activity is inhibited. 25 µM of each glycan substrate was used with 2 U / µL or 0.05 U / µL of α1-6-mannosidase for G2T or Man-α1-6-Man tetrasaccharide, respectively, and incubated for 2 hrs at 37 °C. Reaction byproduct peaks associated with blank or dMNJ sample injections are denoted with asterisks. (C) Treatment of LacZ β1-4-galactosidase with 10 or 100 nmol of dMNJ inhibitor / unit of enzyme (0.05 U / µL) for 4 h at 37 °C showing similar hydrolysis profile of G2T cleavage. Allolactose byproduct peak is denoted with an asterisk.

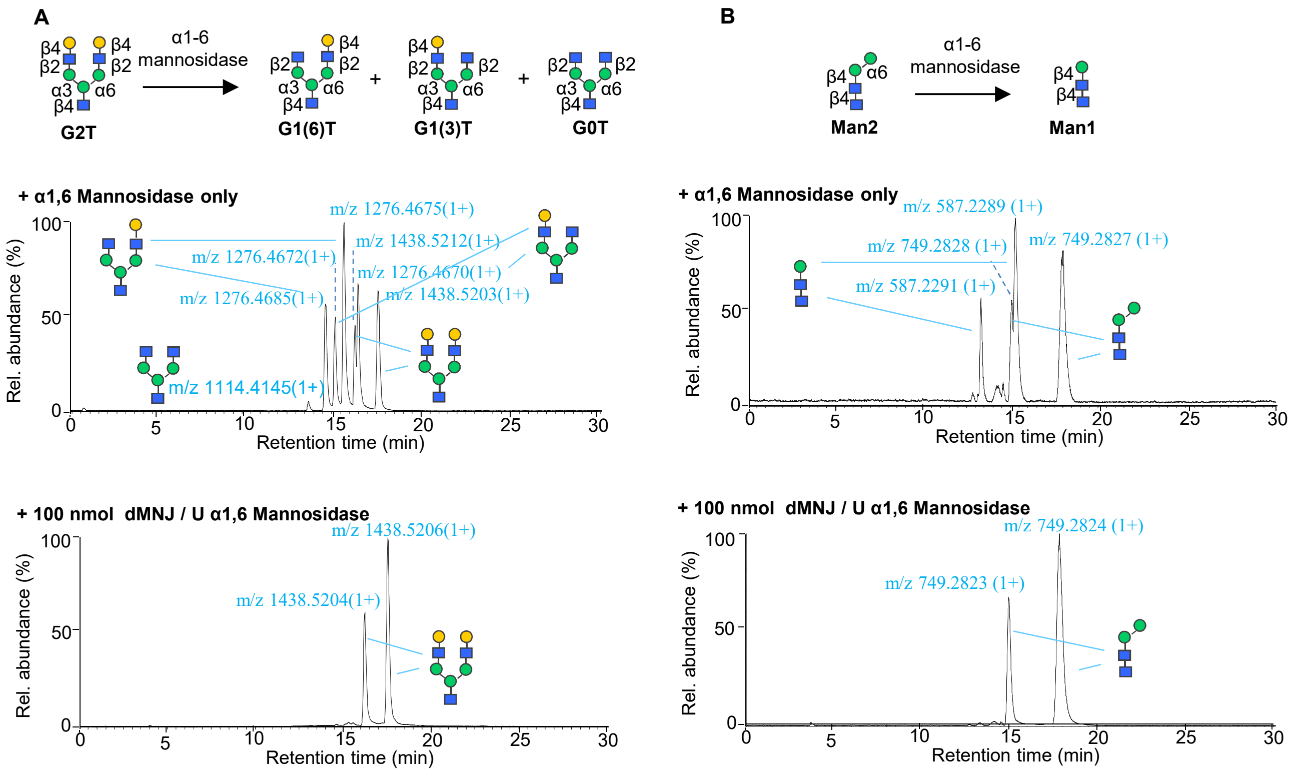

Figure S8. Structural identification of glycan substrates reacted with X. manihotis α1-6 mannosidase under the treatments with and without 1-deoxymannojirimycin (dMNJ). (A) digalactosylated G2T glycan 10, and (B) Man-α1-6-Man tetrasaccharide. α- and β-anomeric peaks are observed in each glycan component by PGC LC MS/MS analyses.

```
XmGH MHTRRDILQLLGAS--AGAGLLAGALPA-----LAA--APAAGTSSTTGRFVSKRPPKAQRRFVSKAVEQQI 63
3QRY ------------------------------------------------------------MVYSKEIVREWL 12
3p2c MK--KQ-IKYISAGMLAGMLLCGGELQAS------NR-MTEMHVCLADAIQKDNRPEISNRLFRSNAVEKEI 62
2p0v ------------MNITKTLCLCAALSGAAGVQAMENREFVTQQDNTRVNNYQTNRPEASKRLFVSQEVERQI 60
2NVP -------------------------------------------MSLSTN-------------ELKEIVRKIG 16
3QPF ------------------------------------------------------------MVYSKEIVREWL 12
                                                                     .: *..

XmGH AQVKARI-ADPELAWLFENCYPNTLDTTVETG-TRNGKPDTFVITGDIHAMWLRDSSAQVHPYVPLAKHDPA 133
3QRY DEVAERAKDYPEWVDVFERCYTDTLDNTVEIL----EDGSTFVLTGDIPAMWLRDSTAQLRPYLHVAKRDAL 80
3p2c LRVQKLL-KNAKLAWMFTNCFPNTLDTTVHFRKGSDGKPDTFVYTGDIHAMWLRDSGAQVWPYVQLANSDPE 133
2p0v DHIKQLL-TNAKLAWMFENCFPNTLDTTVHF----DGKEDTFVYTGDIHAMWLRDSGAQVWPYVQLANKDPE 127
2NVP KDLSGKI-EDKKLQELFYNCFINTMDTTVEVS-----EGDAFVITGDIPAMWLRDSTSQVEHYLPFVKEYPE 82
3QPF DEVAERAKDYPEWVDVFERCYTDTLDNTVEIL----EDGSTFVLTGDIPAMWLRDSTAQLRPYLHVAKRDAL 80
       :        :   :* .*: :*:*.**.       . .:** **** ******* :*:  *: ..:

XmGH LRRMFHGLIQRQAACITLDPYANAFLPDGQTQRLKWSLNDITDMKPGVGERKWEIDSLCYPIRIAHEYWRAT 205
3QRY LRQTIAGLVKRQMTLVLKDPYANSFNIEENWKGH--HETDHTDLNGWIWERKYEVDSLCYPLQLAYLLWKET 150
3p2c LKEMLAGVILRQFKCINIDPYANAFNDGAI-PDGHW-MSDLTDMKPELHERKWEIDSLCYPLRLAYHYWKTT 203
2p0v LKKMLAGVINRQFKCINIDPYANAFNMNS--EGGEW-MSDLTDMKPELHERKWEIDSLCYPIRLAYHYWKTT 196
2NVP LKAIFTGLINRQVKCIFIDPYANAFNKEP--NGQKW-DNDITKDSPWVWERKYEIDSLCYPVRLIHKYWKES 151
3QPF LRQTIAGLVKRQMTLVLKDPYANSFNIEENWKGH--HETDHTDLNGWIWERKYEVDSLCYPLQLAYLLWKET 150
     *:  : *:: **   :  *****:*             .* *. .  : ***:*:******::: :  *: :

XmGH GDAAPFDDDWRAAMHVVVKTFREQQRKD-NRGPYVFQRPSPLATETLVLEGYGQPTKPNGMIHSMFRPSDDA 276
3QRY GETSQFDEIFVAATKEILHLWTVEQDHK--NSPYRFVRDTDRKEDTLVNDGFGPDFAVTGMTWSAFRPSDDC 225
3p2c GDASIFNEEWIQAITNVLKTFKEQQRKD-GVGPYKFQRKTERALDTVSNDGLGAPVKPVGLIVSSFRPSDDA 274
2p0v GDASVFSDEWLQAIANVLKTFKEQQRKDDAKGPYRFQRKTERALDTMTNDGWGNPVKPVGLIASAFRPSDDA 268
2NVP GDETFFNDDIKKAFNMIIDLWRVEQYHR-EKSDYSFQRLNCSVTDTLSHEGLGTPVTYTGMTWSGFRPSDDA 222
3QPF GETSQFDEIFVAATKEILHLWTVEQDHK--NSPYRFVRDTDRKEDTLVNDGFGPDFAVTGMTWSAFRPSDDC 220
     *: : *.:    *   ::. :  :* :    . * * * .    :*:  :* *      *:  * ******.

XmGH CVFPLFVPANLFAVTSLRQLATMSTALHRD--AAFAAECTALADEVETATRQFGQQRDADGQAYWAFEVDGF 346
3QRY CQYSYLIPSNMFAVVVLGYVQEIFAALNLADSQSVIADAKRLQDEIQEGIKNYAYTTNSKGEKIYAFEVDGL 292
3p2c TTLQFLVPSNFFAVSSLRKAAEILEKVNKK--TALSKECKDLAQEVETALKKYAVYNHPKYGKIYAFEVDGF 344
2p0v TTFQFLVPSNFFAVTSLRKAAEILNTVNRK--PALAKECTALADEVEKALKKYAVCNHPKYGKIYAFEVDGF 338
2NVP CEYGYLIPANMFAVVALRYISEIAEKVYKD--EELKEKADSLREEIDNAIEKHGKVYKEGFGEVYAYETDGM 292
3QPF CQYSYLIPSNMFAVVVLGYVQEIFAALNLADSQSVIADAKRLQDEIQEGIKNYAYTTNSKGEKIYAFEVDGL 292
          ::*:*:***  *     :   :       .  ..  * :*:: . .:..        . :*:*.**:

XmGH GNQLFIDDANAPGLLSLAYLGCCDRADPVFLRTRQLAWSERNPYFSRGTAAEGVGSPHSGMGTIWPMSIIQY 418
3QRY GNASIMDDPNVPSLLAAPYLGYCSVDDEVYQATRRTILSSENPYFYQGEYASGLGSSHTFYRYIWPIALSIQ 364
3p2c GNHHLMDDANVPSLLAMPYLGDVNVNDPIYQNTRRFVWSEDNPYFFKGKAGEGIGGPHIGYDMVWPMSIMMK 416
2p0v GNQLLMDDANVPSLIALPYLGDVKVTDPIYQNTRKFVWSEDNPYFFKGSAGEGIGGPHIGYDMIWPMSIMMK 410
2NVP GNYNFMDDANVPSLLSIPYLEYKGIEDEVYQNTRKFILSKNNRFFFEGKAAKGIGSPHTPDQYIWHIALSMQ 364
3QPF GNASIMDDPNVPSLLAAPYLGYCSVDDEVYQATRRTILSSENPYFYQGEYASGLGSSHTFYRYIWPIALSIQ 364
     **  ::** *.*.*::  **      * ::  **:   *. * :* .*  ..*:*. *     :* :::

XmGH ALVSDDDAQLRQCLQWLKTTHAGTGFMHEAFHKDNPNTFTRDWFAWANTLFGELIIDLHQRKP-QLLRSA- 487
3QRY GLTTRDKAEKKFLLDQLVACDGGTGVMHESFHVDDPTLYSREWFSWANMMFCELVLDYLDIR--------- 426
3p2c AFTSQNDAEIKTCIKMLMDTDAGTGFMHESFHKDNPKKFTRAWFAWQNTLFGELILKLVNEGKVDLLNSIQ 487
2p0v AFTSQNDAEIKTCIKMLMDTDAGTGFMHESFNKNDPKNFTRAWFAWQNTLFGELILKLVNEGKVDLLNSIQ 481
2NVP GLTTNNQEEIDQLIKLLKETDAGTGYMHEGFHVDDPTKFTRDWFAWSNSLFSHFIYEKVINKK-------- 427
3QPF GLTTRDKAEKKFLLDQLVACDGGTGVMHESFHVDDPTLYSREWFSWANMMFCELVL--------------- 420
     .:.: :. :    :. *   ..*** ***.*: ::*. ::* **:* * :* .::
```

Figure S9. Protein sequence alignment of α1-6 mannosidases in the GH125 family. Multiple sequence alignment is generated using the Clustal Omega program (https://www.ebi.ac.uk/Tools/msa/clustalo/). XmGH125: *Xanthomonas manihotis* α1-6 mannosidase GH125, NCBI accession #WP_017155573.1; Structural proteins in the PDB database: 3QRY; 3P2C; 2p0v; 2NVP; 3QPF.

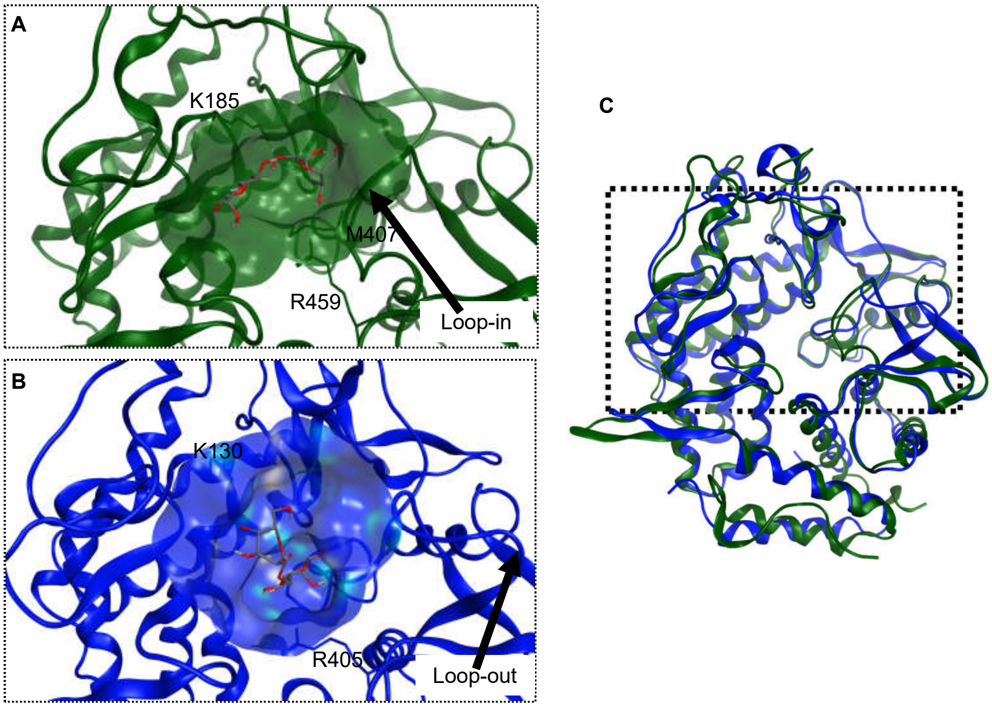

Figure S10. Protein modelling reveals a shallow pocket of α1-6 mannosidase from *X. manihotis* (XmGH125) promoting ligand-receptor promiscuity. (A) Man-α1-6-Man model disaccharide ligand in the 'shallow' enzyme pocket of XmGH125 (green ribbon). 'Loop-in' orientation with extended R459 and M407 is observed to collapse the pocket. (B) Man-α1-6-Man model disaccharide ligand in the 'deep' reference α1-6-mannosidase (PDB: 3QRY) pocket (blue ribbon). R405 is collapsed and the 'loop-out' orientation is observed. (C) Overlay of XmGH125 and reference α1-6-mannosidase (PDB: 3QRY). Docking and figures generated in Molecular Operating Environment 2020, Chemical Computing Group and BioRender, respectively.

Table S1. LC MS/MS identification of the tryptic *Xanthomonas manihotis* α1,6 mannosidase

Peptide	m/z (obs.)	Mr(exp)	Mr(Calc)	PPM	Sequence
1-24	1048.0790(2+)	2094.1434	2094.1433	0.05	-.GLLAGALPALAAAPAAGTSSTTGR.F
1-24	699.0543(3+)	2094.1408	2094.2433	-1.20	-.GLLAGALPALAAAPAAGTSSTTGR.F
4-24	906.4841(2+)	1810.9526	1810.9532	-0.35	(L)AGALPALAAAPAAGTSSTTGR(F)
5-24	870.9658(2+)	1739.9160	1739.9161	-0.06	(A)GALPALAAAPAAGTSSTTGR(F)
5-24	891.9701(2+)	1781.9246	1781.9630	-22.83	VALPALAAAPAAGTSSTTGR(F) *
5-24	891.9701(2+)	1781.9246	1781.9267	-1.25	@GALPALAAAPAAGTSSTTGR(F) (@ = acetylation) *
6-24	842.4550(2+)	1682.8944	1682.8946	-0.12	(G)ALPALAAAPAAGTSSTTGR(F)
25-32	479.7953(2+)	957.5760	957.5760	0.06	R.FVSKRPPK.A
36-50	577.6686(3+)	1729.9841	1729.9839	0.12	R.RFVSKAVEQQIAQVK.A
37-50	787.9484(2+)	1573.8823	1573.8828	-0.30	R.FVSKAVEQQIAQVK.A
37-50	525.6349(3+)	1573.8828	1573.8828	0.04	R.FVSKAVEQQIAQVK.A
37-52	601.3478(3+)	1801.0217	1801.0210	0.39	R.FVSKAVEQQIAQVKAR.I
37-52	451.2628(4+)	1801.0219	1801.0210	0.52	R.FVSKAVEQQIAQVKAR.I
41-50	557.3160(2+)	1112.6175	1112.6190	-1.29	K.AVEQQIAQVK.A
41-52	447.5933(3+)	1339.7580	1339.7572	0.58	K.AVEQQIAQVKAR.I
41-52	670.8865(2+)	1339.7584	1339.7572	0.91	K.AVEQQIAQVKAR.I
51-79	1118.5408(3+)	3352.6005	3352.6034	-0.87	K.ARIADPELAWLFENCYPNTLDTTVETGTR.N
53-79	1042.8280(3+)	3125.4622	3125.4652	-0.96	R.IADPELAWLFENCYPNTLDTTVETGTR.N
53-79	1563.7390(2+)	3125.4635	3125.4652	-0.55	R.IADPELAWLFENCYPNTLDTTVETGTR.N
80-98	724.3732(3+)	2170.0979	2170.0994	-0.69	R.NGKPDTFVITGDIHAMWLR.D
80-98	1086.0559(2+)	2170.0973	2170.0994	-0.96	R.NGKPDTFVITGDIHAMWLR.D
80-98	543.5325(4+)	2170.1008	2170.0994	0.66	R.NGKPDTFVITGDIHAMWLR.D
99-112	756.3956(2+)	1510.7767	1510.7780	-0.85	R.DSSAQVHPYVPLAK.H
99-112	504.6000(3+)	1510.7783	1510.7780	0.20	R.DSSAQVHPYVPLAK.H
99-118	734.3878(3+)	2200.1415	2200.1389	1.17	R.DSSAQVHPYVPLAKHDPALR.R
113-118	354.6927(2+)	707.3708	707.3715	-0.98	K.HDPALR.R
113-119	432.7436(2+)	863.4726	863.4726	0.07	K.HDPALRR.M
119-127	386.5500(3+)	1156.6282	1156.6288	-0.45	R.RMFHGLIQR.Q
119-127	579.3215(2+)	1156.6284	1156.6288	-.032	R.RMFHGLIQR.Q
120-127	501.2709(2+)	1000.5273	1000.5276	-0.40	R.MFHGLIQR.Q
128-149	1225.5909(2+)	2449.1673	2449.1696	-0.93	R.QAACITLDPYANAFLPDGQTQR.L
128-149	817.3965(3+)	2449.1676	2449.1696	-0.81	R.QAACITLDPYANAFLPDGQTQR.L
128-151	897.7895(3+)	2690.3466	2690.3486	-0.73	R.QAACITLDPYANAFLPDGQTQRLK.W
150-167	1030.0355(2+)	2058.0565	2058.0568	-0.15	R.LKWSLNDITDMKPGVGER.K
150-167	687.0261(3+)	2058.0565	2058.0568	-0.13	R.LKWSLNDITDMKPGVGER.K
150-167	515.5215(4+)	2058.0568	2058.0568	0.01	R.LKWSLNDITDMKPGVGER.K
150-168	547.5453(4+)	2186.1521	2186.1517	0.14	R.LKWSLNDITDMKPGVGERK.W
152-167	909.4453(2+)	1816.8761	1816.8778	-0.94	K.WSLNDITDMKPGVGER.K
152-167	606.6326(3+)	1816.8761	1816.8778	-0.94	K.WSLNDITDMKPGVGER.K
152-168	649.3312(3+)	1944.9717	1944.9727	-0.52	K.WSLNDITDMKPGVGERK.W
152-168	487.2503(2+)	1944.9721	1944.9727	-0.31	K.WSLNDITDMKPGVGERK.W
168-179	790.4000(2+)	1578.7854	1578.7864	-0.67	R.KWEIDSLCYPIR.I
168-179	527.2691(3+)	1578.7855	1578.7864	-0.60	R.KWEIDSLCYPIR.I
168-186	634.5707(4+)	2534.2536	2534.2528	0.31	R.KWEIDSLCYPIRIAHEYWR.A
169-179	726.3527(2+)	1450.6908	1450.6915	-0.48	K.WEIDSLCYPIR.I
169-179	484.5710(3+)	1450.6913	1450.6915	-0.12	K.WEIDSLCYPIR.I
180-186	487.7454(2+)	973.4763	973.4770	-0.64	R.IAHEYWR.A
187-199	718.8072(2+)	1435.5999	1435.6004	-0.34	R.ATGDAAPFDDDWR.A
187-199	479.5402(3+)	1435.5989	1435.6004	-1.05	R.ATGDAAPFDDDWR.A
187-207	1136.5443(2+)	2271.0741	2271.0743	-0.09	R.ATGDAAPFDDDWRAAMHVVVK.T
187-207	758.0318(3+)	2271.0736	2271.0743	-0.31	R.ATGDAAPFDDDWRAAMHVVVK.T
200-207	427.7492(2+)	853.4839	853.4844	-0.54	R.AAMHVVVK.T
208-214	482.7514(2+)	963.4883	963.4886	-0.32	K.TFREQQR.K
208-215	364.8684(3+)	1091.5832	1091.5836	-0.32	K.TFREQQRK.D
216-225	626.3144(2+)	1250.6143	1250.6156	-1.01	K.DNRGPYVFQR.P
219-244	950.1663(3+)	2847.4771	2847.4807	-1.25	R.GPYVFQRPSPLATETLVLEGYGQPTK.P
219-254	1005.5129(4+)	4018.0227	4018.0233	-0.17	R.GPYVFQRPSPLATETLVLEGYGQPTKPNGMIHSMFR.P
226-254	793.9004(4+)	3171.5727	3171.5733	-0.18	R.PSPLATETLVLEGYGQPTKPNGMIHSMFR.P
255-277	1268.6476(2+)	2535.2806	2535.2832	-1.02	R.PSDDACVFPLFVPANLFAVTSLR.Q
278-288	410.2202(3+)	1227.6389	1227.6394	-0.37	R.QLATMSTALHR.D
278-288	614.8270(2+)	1227.6394	1227.6394	0.01	R.QLATMSTALHR.D

278-308	1107.8660(3+)	3320.5761	3320.5765	-0.13	R.QLATMSTALHRDAAFAAECTALADEVETATR.Q
278-308	831.1516(4+)	3320.5771	3320.5765	0.18	R.QLATMSTALHRDAAFAAECTALADEVETATR.Q
289-308	1056.4812(2+)	2110.9478	2110.9477	0.07	R.DAAFAAECTALADEVETATR.Q
289-308	704.6565(3+)	2110.9476	2110.9477	-0.02	R.DAAFAAECTALADEVETATR.Q
289-314	952.7780(3+)	2855.3122	2855.3144	-0.77	R.DAAFAAECTALADEVETATRQFGQQR.D
309-314	382.1959(2+)	762.3772	762.3773	-0.16	R.QFGQQR.D
315-354	1107.0024(4+)	4423.9807	4423.9790	0.36	R.DADGQAYWAFEVDGFGNQLFIDDANAPGLLSLAYLGCCDR.A
315-354	1475.6677(3+)	4423.9813	4423.9790	0.52	R.DADGQAYWAFEVDGFGNQLFIDDANAPGLLSLAYLGCCDR.A
355-361	409.2320(2+)	816.4494	816.4494	-0.02	R.ADPVFLR.T
362-370	573.8046(2+)	1145.5946	1145.5941	0.40	R.TRQLAWSER.N
362-376	637.6589(3+)	1909.9548	1909.9547	0.05	R.TRQLAWSERNPYFSR.G
362-376	478.4960(4+)	1909.9550	1909.9547	0.17	R.TRQLAWSERNPYFSR.G
364-370	445.2300(2+)	888.4455	888.4453	0.14	R.QLAWSER.N
364-376	827.4101(2+)	1652.6056	1652.8059	-0.16	R.QLAWSERNPYFSR.G
364-376	551.9425(3+)	1652.8057	1652.8059	-0.14	R.QLAWSERNPYFSR.G
377-412	1244.2658(3+)	3729.7754	3729.7767	-0.34	R.GTAAEGVGSPHSGMGTIWPMSIIQYALVSDDDAQLR.Q
377-412	933.4510(4+)	3729.7751	3729.7767	-0.43	R.GTAAEGVGSPHSGMGTIWPMSIIQYALVSDDDAQLR.Q
413-419	488.2577(2+)	974.5008	974.5008	0.01	R.QCLQWLK.T
420-434	836.3885(2+)	1670.7624	1670.7624	0.03	K.TTHAGTGFMHEAFHK.D
420-434	557.9281(3+)	1670.7625	1670.7624	0.06	K.TTHAGTGFMHEAFHK.D
420-434	418.6979(4+)	1670.7625	1670.7624	0.09	K.TTHAGTGFMHEAFHK.D
420-442	873.0717(3+)	2616.1932	2616.1928	0.12	K.TTHAGTGFMHEAFHKDNPNTFTR.D
420-442	655.0557(4+)	2616.1935	2616.1926	0.27	K.TTHAGTGFMHEAFHKDNPNTFTR.D
435-442	482.7280(2+)	963.4414	963.4410	0.45	K.DNPNTFTR.D
435-462	1130.8926(3+)	3389.6559	3389.6582	-0.66	K.DNPNTFTRDWFAWANTLFGELIIDLHQR.K
435-462	848.4213(4+)	3389.6562	3389.6582	-0.57	K.DNPNTFTRDWFAWANTLFGELIIDLHQR.K
443-462	1223.1207(2+)	2444.2269	2444.2277	-0.33	R.DWFAWANTLFGELIIDLHQR.K
443-462	815.7496(3+)	2444.2271	2444.2277	-0.26	R.DWFAWANTLFGELIIDLHQR.K
463-468	377.7503(2+)	753.4860	753.4861	-0.13	R.KPQLLR.S

* Mass accuracy measurements and MS/MS analyses identified the N-terminal residue of peptide 5-24 is acetylated glycine rather than valine.
